# Supplementary material for: A Critical View of the Application of the APEX Software (Aqueous Photochemistry of Environmentally-Occurring Xenobiotics) to Predict Photoreaction Kinetics in Surface Freshwaters
Source: Molecules. 2019 Dec 18;25(1):9. doi: 10.3390/molecules25010009 (PMC7017383; doi:10.3390/molecules25010009)
Supplement: Supplementary file 1 [file molecules-25-00009-s001.zip › 3.2.4_gcc-4.4.0.zip!/3.2.4_gcc-4.4.0/3.2.4_gcc-4.4.0/share/octave/packages/fixed-0.7.10/doc/fixed.pdf]

Fixed Point Toolbox for Octave

Version 0.7.4
January 2004

David Bateman
Laurent Mazet

Copyright © 2004 Motorola Inc

Permission is granted to make and distribute verbatim copies of this manual provided the copyright notice and this permission notice are preserved on all copies.

Permission is granted to copy and distribute modified versions of this manual under the conditions for verbatim copying, provided that the entire resulting derived work is distributed under the terms of a permission notice identical to this one.

Permission is granted to copy and distribute translations of this manual into another language, under the same conditions as for modified versions.

Table of Contents

1 The Basics of the Fixed Point Types 1

1.1 The License used with this Package 1
1.2 Fixed Point Representation 1
1.3 Creating Fixed Point Numbers 2
1.4 Overflow Behavior of Fixed Point Numbers 4
1.5 Fixed Point Built-in Variables 5
1.6 Accessing Internal Fields 6
1.7 Function Overloading 7
1.8 Putting it all Together 8
1.9 Problems of Precision in Calculations 8
1.10 Lookup Tables 9
1.11 Known Problems 9

2 Using the fixed-point type in C++ and oct-files 12

2.1 The `FixedPoint` Base Class 12
2.1.1 `FixedPoint` Constructors 12
2.1.2 `FixedPoint` Specific Methods 13
2.1.3 `FixedPoint` Operators 14
2.1.4 `FixedPoint` Functions 16
2.2 The `FixedPointComplex` Base Class 17
2.2.1 `FixedPointComplex` Constructors 17
2.2.2 `FixedPointComplex` Specific Methods 20
2.2.3 `FixedPointComplex` Operators 21
2.2.4 `FixedPointComplex` Functions 22
2.3 The Derived Classes using the Octave Template Classes . . . 24
2.3.1 `FixedMatrix` class 24
2.3.2 `FixedRowVector` class 27
2.3.3 `FixedColumnVector` class 29
2.3.4 `FixedComplexMatrix` class 32
2.3.5 `FixedComplexRowVector` class 36
2.3.6 `FixedComplexColumnVector` class 40
2.4 The Upper Level Octave Classes 44
2.5 Writing Oct-files with the Fixed Point Type 45
2.5.1 Using C++ Templates in Oct-files 45
2.5.2 Specific Problems of Oct-files using Fixed Point . . 46
2.5.3 Specific points to note when using Oct-files and Cygwin 46
2.5.4 A Simple Example of an Oct-file 47

3 Fixed Point Type Applied to Real Signal Processing Example 49

4 **Function Reference** 51
4.1 Functions by Category 51
4.1.1 Fixed Point Operators 51
4.1.2 Fixed Point Variables 51
4.1.3 Fixed Point Utility Functions 51
4.1.4 Fixed Point Functions 52
4.1.5 Examples 53
4.2 Functions Alphabetically 53
4.2.1 concat 53
4.2.2 create_lookup_table 53
4.2.3 display_fixed_operations 54
4.2.4 fabs 54
4.2.5 fangle 54
4.2.6 farg 54
4.2.7 fatan2 54
4.2.8 fceil 54
4.2.9 fconj 54
4.2.10 fcos 55
4.2.11 fcosh 55
4.2.12 fcumprod 55
4.2.13 fcumsum 55
4.2.14 fdiag 55
4.2.15 fexp 56
4.2.16 ffft 56
4.2.17 ffloor 56
4.2.18 fifft 56
4.2.19 fimag 56
4.2.20 fixed 57
4.2.21 fixed_inc 57
4.2.22 fixed_point_count_operations 57
4.2.23 fixed_point_debug 57
4.2.24 fixed_point_library_version 58
4.2.25 fixed_point_version 58
4.2.26 fixed_point_warn_overflow 58
4.2.27 fixedpoint 58
4.2.28 float 59
4.2.29 flog 59
4.2.30 flog10 59
4.2.31 fprod 59
4.2.32 freal 59
4.2.33 freshape 60
4.2.34 fround 60
4.2.35 fsin 60
4.2.36 fsinh 60
4.2.37 fsort 61
4.2.38 fsqrt 61
4.2.39 fsum 61
4.2.40 fsumsq 61

4.2.41 ftan 62
4.2.42 ftanh 62
4.2.43 isfixed 62
4.2.44 lookup_table 62
4.2.45 reset_fixed_operations 62

1 The Basics of the Fixed Point Types

When implementing algorithms in hardware, it is common to reduce the accuracy of the representation of numbers to a smaller number of bits. This allows much lower complexity in the hardware, at the cost of accuracy and potential overflow problems. Such representations are known as fixed point.

This toolbox supplies a fixed point type that allows Octave to model the effects of such a reduction in accuracy of the representation of numbers. The major advantage of this toolbox is that with correctly written Octave scripts, the same code can be used to test both fixed and floating point representations of numbers.

The authors have tried to take all care to ensure the correct functionality of this package. However, as this code is relatively recent we expect that there will be a certain number of problems. We welcome all reports of bugs, etc or even success stories. The authors can be contacted at the e-mail address David.Bateman@motorola.com.

1.1 The License used with this Package

The license used with this package is the GNU General Public License, a copy of which is distributed with this package in the file 'COPYING'. Some commercial users have seemed quite concerned about the use of software licensed under the GPL in their development. To ease these concerns, the authors state in clear English the terms of the GPL allow that

1. Any algorithm developed with this package remains the sole property of the party developing the algorithm.
2. Changes can be made to the fixed point package, with the constraint that if you supply a version of the fixed point package to another party it must equally be covered by the terms of the GPL.

We believe that there is little reason to make proprietary changes to the fixed point package itself. So this clear distinction between the fixed point code itself and algorithms developed with it means that there should be little concern for a use of this package in the development of a proprietary algorithms.

Proprietary changes to the fixed point package itself are possible. The GPL only comes into play in if you distribute the fixed point package with these changes. The algorithms developed on these modified versions of the fixed point package remain proprietary in all cases.

1.2 Fixed Point Representation

Fixed point numbers can be represented digitally in several manners, including *sign-magnitude*, *ones-complement* and *twos-complement*. The most commonly used technique is *twos-complement* due to the easier implementation of certain operations in this representation. As such this toolbox uses the *twos-complement* representation of numbers

All fixed point objects in this toolbox are represented by an *int* that is used in the following manner

1 bit representing the sign,

is bits representing the integer part of the number, and

ds bits representing the decimal part of the number.

The numbers that can then be represented are then given by

$$-2^{is} \leq x \leq 2^{is} - 2^{-ds}$$

and the distance between two values of x that are not represented by the same fixed point value is

$$2^{-ds}$$

.

The number of bits that can be used in the representation of the fixed point objects is determined by the number of bits in an *int* on the platform. Valid values include 32- and 64-bits. To avoid issues with overflows in additions, one of these bits can not be used. Therefore valid values of *is* and *ds* are given by

$$0 < (is + ds) \leq n - 2$$

where n is either 32 or 64, depending on the number of bits in an *int*. It should be noted that given the above criteria it is possible to create a fixed point representation that lacks a representation of the number 1. This makes the implementation of certain operators difficult, and so the valid representations are further limited to

$$0 < (is, ds, is + ds) \leq n - 2$$

This does not mean that other numbers can not be represented by this toolbox, but rather that the numbers must be scaled prior to their being represented.

This toolbox allows both fixed point real and complex scalars to be represented, as well as fixed point real and complex matrices. The real and imaginary parts of the fixed point number and each element of a fixed point matrix has its own fixed point representation.

1.3 Creating Fixed Point Numbers

Before using a fixed point type, some variables must be created that use this type. This is done with the function *fixed*. The function *fixed* can be used in several manners, depending on the number and type of the arguments that are given. It can be used to create scalar, complex, matrix and complex matrix values of the fixed type.

The generic call to *fixed* is `fixed(`*is*`,`*ds*`,`*f*`)`, where the variables are

is
: The number of bits to use in the representation of the integer part of the fixed point value. This can be either a real or complex value, and can be either a scalar or a matrix. As the fixed point representation of complex values uses separate representations for the real and imaginary parts, a complex value of *is* gives the representation of the real and imaginary parts separately. *is* must contain only integer or complex integer values greater than zero, and less than 30 or 62 as discussed in the previous section.

ds Similarly to *is*, *ds* represents the number of bits in the decimal part of the fixed point representation. The same conditions as for *is* apply to *ds*

f This variable can be either a scalar, complex, matrix or complex matrix of values that will be converted to a fixed point representation. It can equally be another fixed point value, in which case *fixed* has the effect of changing the representation of *f* to another representation given by *is* and *ds*.

If matrices are used for *is*, *ds*, or *f*, then the dimensions of all of the matrices must match. However, it is valid to have *is* or *ds* as scalar values, which will be expanded to the same dimension as the other matrices, before use in the conversion to a fixed point value. The variable *f* however, must be a matrix if either *is* or *ds* is a matrix.

The most basic use of the function *fixed* can be seen in the example

```
octave:1> a = fixed(7,2,1)
ans = 1
octave:2> isfixed(a)
ans = 1
octave:3> whos a
*** local user variables:

prot  type                       rows   cols  name
====  ====                       ====   ====  ====
 rwd  fixed scalar                  1      1  a
```

which demonstrates the creation of a real scalar fixed point value with 7 bits of precision in the integer part, 2 bits in the decimal part and the value 1. The function *isfixed* can be used to identify whether a variable is of the fixed point type or not. Equally, using the *whos* function allows the variable to be identified as "fixed scalar".

Other examples of valid uses of *fixed* are

```
octave:1> a = fixed(7, 2, 1);
octave:2> b = fixed(7, 2+1i, 1+1i);
octave:3> c = fixed(7, 2, 255*rand(10,10) - 128);
octave:4> is = 3*ones(10,10) + 4*eye(10);
octave:5> d = fixed(is, 1, eye(10));
octave:6> e = fixed(7, 2, 255*rand(10,10)-128 +
> 1i*(255*rand(10,10)-128));
octave:7> whos

*** local user variables:

prot  type                       rows   cols  name
====  ====                       ====   ====  ====
 rwd  fixed scalar                  1      1  a
 rwd  fixed complex                 1      1  b
 rwd  fixed matrix                 10     10  c
 rwd  fixed matrix                 10     10  d
 rwd  fixed complex matrix         10     10  e
```

With two arguments given to *fixed*, it is assumed that *f* is zero or a matrix of zeros, and so *fixed* called with two arguments is equivalent to calling with three arguments with the third arguments being zero. For example

```
octave:1> a = fixed([7,7], [2,2], zeros(1,2));
octave:2> b = fixed([7,7], [2,2]);
octave:3> assert(a == b);
```

Called with a single argument *fixed*, and a fixed point argument, `b = fixed(a)` is equivalent to `b = a`. If *a* is not itself fixed point, then the integer part of *a* is used to create a fixed point value, with the minimum number of bits needed to represent it. For example

```
octave:1> b = fixed(1:4);
```

creates a fixed point row vector with 4 values. Each of these values has the minimum number of bits needed to represent it. That is b(1) uses 1 bit to represent the integer part, b(2:3) use 2 bits and b(4) uses 3 bits. The single argument used with *fixed* can equally be a complex value, in which case the real and imaginary parts are treated separately to create a composite fixed point value.

1.4 Overflow Behavior of Fixed Point Numbers

When converting a floating point number to a fixed point number the overflow behavior of the fixed point type is such that it implements clipping of the data to the maximum or minimum value that is representable in the fixed point type. This effectively simulates the behavior of an analog to digital conversion. For example

```
octave:1> a = fixed(7,2,200)
a = 127.75
octave:2> a = fixed(7,2,-200)
a = -128
```

However, the overflow behavior of the fixed point type is distinctly different if the overflow occurs within a fixed point operation itself. In this case the excess bits generated by the overflow are dropped. For example

```
octave:1> a = fixed(7,2,127) + fixed(7,2,2)
a = -127
octave:2> a = fixed(7,2,-127) + fixed(7,2,-2)
a = 127
```

The case where the representation of the fixed point object changes is different again. In this case the sign is maintained, while the most-significant bits of the representation are dropped. For example

```
octave:1> a = fixed(6, 2, fixed(7, 2, -127.25))
a = -63.25
octave:2> a = fixed(6, 2, fixed(7, 2, 127.25))
a = 63.25
octave:3> a = fixed(7, 1, fixed(7, 2, -127.25))
a = -127.5
octave:4> a = fixed(7, 1, fixed(7, 2, 127.25))
a = 127
```

In addition to the overflow issue discussed above, it is important to take into account what happens when an operator is used on two fixed point values with different representations. For example

```
octave:1> a = fixed(7,2,1);
octave:2> b = fixed(6,3,1);
octave:3> c = a + b;
octave:4> fprintf("%d integer, and %d decimal bits\n", c.int, c.dec);
7 integer, and 3 decimal bits
```

as can be seen the fixed point value is promoted to have an output fixed point representation such that `c.int = max(a.int,b.int)` and `c.dec = max(a.dec,b.dec)`. If this promotion causes the maximum number of bits in a fixed point representation to be exceeded, then an error will occur.

1.5 Fixed Point Built-in Variables

After the fixed point type is first used, four variables are initialized. These are

fixed_point_version
: The version number of the fixed point code

fixed_point_warn_overflow
: If non-zero warn of fixed point overflows. The default is 0.

fixed_point_count_operations
: If non-zero count number of fixed point operations, for later complexity analysis

fixed_point_debug
: If non-zero keep a copy of fixed point value to allow easier debugging with gdb

and they can be accessed as normal Octave built-in variables. The variable `fixed_point_version` can be used to create tests in the users code, to work-around any eventual problems in the fixed point type. For example

```
if (strcmp(fixed_point_version, "0.6.0"))
  a = fixed([a.int, b.int], [a.dec, b.dec],
            [a.x, b.x]);
else
  a = concat(a, b);
endif
```

although this is not a real example, since both versions of the above code work with the released version of the fixed point type.

When optimizing the number of bits in a fixed point type, it is normal to expect overflows to occur, causing errors in the calculations which due to the implementation have little effect on the end result of the system. However, it is sometimes useful to know exactly where overflows are happening or not. A non-zero value of variable `fixed_point_warn_overflow` permits the errors conditions in fixed point operations to cause a warning message to be printed by octave. The default behavior is to have `fixed_point_warn_overflow` be 0.

The octave fixed point type can keep track of all of the fixed point operations and their type. This is very useful for a simple complexity analysis of the algorithms. To allow the fixed point type to track operations the variable `fixed_point_count_operations` must be

non-zero. The count of operations can then be reset with the *reset_fixed_operations*, and the number of operations since the last reset can be given by the *display_fixed_operations* function.

The final in-built variable of the fixed point type is `fixed_point_debug`. In normal operation this variable is of no use. However setting it to a non-zero value causes a copy of the floating point representation of a fixed point value to be stored internally. This makes debugging code using the fixed point type significantly easier using gdb.

1.6 Accessing Internal Fields

Once a variable has been defined as a fixed point object, the parameters of the field of this structure can be obtained by adding a suffix to the variable. Valid suffixes are '.x', '.i', '.sign', '.int' and '.dec', which return

.x The floating point representation of the fixed point number

.i The internal integer representation of the fixed point number

.sign The sign of the fixed point number

.int The number of bits representing the integer part of the fixed point number

.dec The number of bits representing the decimal part of the fixed point number

As each fixed point value in a matrix can have a different number of bits in its representation, these suffixes return objects of the same size as the original fixed point object. For example

```
octave:1> a = [-3:3];
octave:2> b = fixed(7,2,a);
octave:3> b.sign
ans =

  -1  -1  -1   0   1   1   1
octave:4> b.int
ans =

  7  7  7  7  7  7  7
octave:5> b.dec
ans =

  2  2  2  2  2  2  2
octave:5> c = b.x;
octave:6> whos

*** local user variables:

prot  type                       rows   cols  name
====  ====                       ====   ====  ====
 rwd  matrix                        1      7  a
 rwd  fixed matrix                  1      7  b
 rwd  matrix                        1      7  c
```

The suffixes '.int' and '.dec' can also be used to change the internal representation of a fixed point value. This can result in a loss of precision in the representation of the fixed point value, which models the same process as occurs in hardware. For example

```
octave:1> b = fixed(7,2,[3.25, 3.25]);
octave:2> b(1).dec = [0, 2];
b =

      3  3.25
```

However, the value itself should not be changed using the suffix '.x'. For instance

```
octave:3> b.x = [3, 3];
error: can not directly change the value of a fixed structure
error: assignment failed, or no method for ‘fixed matrix = matrix’
error: evaluating assignment expression near line 3, column 6
```

1.7 Function Overloading

An important consideration in the use of the fixed point toolbox is that many of the internal functions of Octave, such as *diag*, can not accept fixed point objects as an input. This package therefore uses the *dispatch* function of Octave-Forge to *overload* the internal Octave functions with equivalent functions that work with fixed point objects, so that the standard function names can be used. However, at any time the fixed point specific version of the function can be used by explicitly calling its function name. The correspondence between the internal function names and the fixed point versions is as follows

Normal	Specific	Normal	Specific	Normal	Specific
abs	fabs	atan2	fatan2	ceil	fceil
conj	fconj	cos	fcos	cosh	fcosh
cumprod	fcumprod	cumsum	fcumsum	diag	fdiag
exp	fexp	floor	ffloor	imag	fimag
log10	flog10	log	flog	prod	fprod
real	freal	reshape	freshape	round	fround
sin	fsin	sinh	fsinh	sqrt	fsqrt
sum	fsum	sumsq	fsumsq	tan	ftan
tanh	ftanh				

The version of the function that is chosen is determined by the first argument of the function. So, considering the *atan2* function, if the first argument is a *Matrix*, then the normal version of the function is called regardless of whether the other argument of the function is a fixed point objects or not.

Many other Octave functions work correctly with fixed point objects and so overloaded versions are not necessary. This includes such functions as *size* and *any*.

It is also useful to use the '.x' option discussed in the previous section, to extract a floating point representation of the fixed point object for use with some functions.

1.8 Putting it all Together

Now that the basic functioning of the fixed point type has been discussed, it is time to consider how to put all of it together. For the list of functions and operators available for the fixed point type see Chapter 4 [Function Reference], page 51

The main advantage of this fixed point type over an implementation of specific fixed point code, is the ability to define a function once and use as either fixed or floating point. Consider the example

```
function [b, bf] = testfixed(is,ds,n)
a = randn(n,n);
af = fixed(is,ds,a);
b = myfunc(a,a);
bf = myfunc(af,af);
endfunction

function y = myfunc(a,b)
y = a + b;
endfunction
```

In this case `b` and `bf` will be returned from the function *testfixed* as floating and fixed point types respectively, while the underlying function *myfunc* does not explicitly define that it uses a fixed point type. This is a major advantage, as it is critical to understand the loss of precision in an algorithm when converting from floating to fixed point types for an optimal hardware implementation. This mixing of functions that treat both floating and fixed point types can even apply to Oct-files (see Section 2.5 [Oct-files], page 45).

The main limitation to the above is the use of the concatenation operator, such as `[a,b]`, that is hard-coded in versions of Octave prior to 2.1.58 and is thus not aware of the fixed-point type. Therefore, such code should be avoided in earlier versions of Octave and the function *concat* supplied with this package used instead.

1.9 Problems of Precision in Calculations

When dimensioning the fixed point variables, care must be taken so that all intermediate operations don't cause a loss in the precision. This can occur with any operator or function that takes a large argument and gives a small result. Minor variations in the initial argument can result in large changes in the final result.

For instance, consider the *log* operator, in the example

```
octave:1> a = fixed(7,2,5.25);
octave:2> b = exp(log(a))
b = 4.25
```

The logarithm `log(a)` is 1.65, which is rounded to 1.5. The exponential `exp(log(a))` is then 4.48 which is rounded to 4.25.

A particular case in point is the power operator for complex number, which is implemented by the standard C++ class as

$$x^y = \exp(y \log(x))$$

Unless a large decimal precision is specified for this operator, the results will be wildly different than expected. For example

```
octave:1> fixed(7,2,4*1i) ^ fixed(7,2,1)
ans = 0.000 + 2.250i
octave:2> fixed(7,5,4*1i) ^ fixed(7,5,1)
ans = 0.000000 + 3.812500i
```

If the user chooses to use certain functions and operators, it is their responsibility to understand the implementation of the these operators, as used by their compilers to ensure the desired behavior. Alternatively, the user is recommended to implement certain operations as lookup tables, rather than use the built-in operator or function. This is how such general functions are implemented in hardware and so this is not a significant problem.

1.10 Lookup Tables

It is common to implement complex functions in hardware by an equivalent lookup table. This has the advantage of speed, saving on the complexity of a full implementation of certain functions, and avoiding rounding errors if the complex function is implemented as a combination of sub-functions. The disadvantage is that the lookup requires the use of a read-only memory in hardware. Due to size limitations on this memory it might not be possible to represent all possible values of a function.

This section discusses the use of lookup tables with the fixed point type. It is assumed that the function *lookup* of Octave-forge is installed. The easiest way to explain the use of a fixed point lookup table is to discuss an example. Consider a fixed point value in the range `-pi:pi`, and we wish to represent the sine function in this range. The creation of the lookup table can then be performed as follows.

```
octave:1> is = 2; ds = 6;
octave:2> x = [-3.125:0.125:3.125];  % 3.125 ~ pi
octave:3> y = sin(x);
octave:4> table_float = create_lookup_table(x, y);
octave:5> table_fixed = create_lookup_table(fixed(is,ds,x),
>                             fixed(is,ds,y));
```

A real implementation of this function in hardware might use to the symmetry of the sine function to only require the lookup table for `[0:pi/2]` to be stored. However, for simulation there is little reason to introduce this complication.

To evaluate the value of the function use the lookup table created by *create_lookup_table*, the function *lookup_table* is then used. This function can either be used to give the closest evalued value below the desired value, or it can be used to interpolate the table as might be done in hardware. For example

```
octave:6> x0 = [-pi:0.01:pi];
octave:7> y0 = sin(x);
octave:8> y1 = lookup_table(table_float, x0, "linear");
octave:9> y2 = lookup_table(table_fixed, fixed(is,ds,x0), "linear");
```

1.11 Known Problems

Before reporting a bug compare it to this list of known problems

Concatenation

For versions of Octave prior to 2.1.58, the concatenation of fixed point objects returns a Matrix type. That is `[fixed(7,2,[1, 2]), fixed(7,2,3)]` returns a matrix when it should return another fixed point matrix. This problem is due to the implementation of matrix concatenation in earlier versions of Octave being hard-coded for the basic types it knows rather than being expandable.

The workaround is to explicitly convert the returned value back to the correct fixed point object. For instance

```
octave:1> a = fixed(7,2,[1,2]);
octave:2> b = fixed(7,2,3);
octave:3> c = fixed([a.int, b.int], [a.dec, b.dec],
>            [a.x, b.x]);
```

Alternatively, use the supplied function *concat* that explicitly performs the above above, but can also be used for normal matrices.

Since Octave version 2.1.58, `[fixed(7,2,[1,2]),fixed(7,2,3)]` returns another fixed point object as expected.

Saving fixed point objects

Saving of fixed point variables is only implemented in versions of Octave later than 2.1.53. If you are using a recent version of Octave then saving a fixed point variable is as simple as

```
octave:2> save a.mat a
```

where *a* is a fixed point variable. To reload the variable within octave, the fixed point types must be installed prior to a call to *load*. That is

```
octave:1> dummy = fixed(1,1);
octave:2> load a.mat
```

With versions of octave later than 2.1.53, fixed point variables can be saved in the octave binary and ascii formats, as well as the HDF5 format. If you are using an earlier version of octave, you can not directly save a fixed point variable. You can however save the information it contains and reconstruct the data afterwards by doing something like

```
octave:2> x = a.x; is = a.int; ds = a.dec;
octave:3> save a.mat x is ds;
```

where *a* is a fixed point object.

Some functions and operators return very poor results

Please read the previous section. Also if the problem manifests when using complex arguments, try to understand your compilers constructor of complex operators and functions from the base fixed point operators and functions. The relevant file for the gcc 3.x versions of the compiler can be found in '`/usr/include/g++-v3/bits/std_complex.h`'.

Function *foo* returns fixed point types while *bar*

does not? If the existing functions, written as m-files, respect the use (or rather non-use) of the concatentaion operator, then these functions will operate correctly. However, many functions don't and thus will return a floating type for a fixed point input, when run on versions of Octave earlier than 2.1.58. All

functions should be checked by the user for their correct operation before using them.

Additionally, existing oct-files will not operate correctly with fixed point inputs, as they are not aware of the fixed point type and will just extract the floating point value to operate on.

A third class of function are the inbuilt functions like *any*, *size*, etc. As the fixed point type includes the underlying functions for these to work correctly, they give the correct result even though there is no corresponding fixed point specific version of these functions.

Why is my code so slow when using fixed point

This is due to several reasons, firstly the normal functions in octave use optimized libraries to accelerate their operation. This is not possible when using fixed point.

Also there is no fixed point type native to the machines Octave runs on. This naturally makes the fixed point type slow, due to the fact that the fixed point operators check for overflows, etc at all steps in the operation and act accordingly. This is particularly true in the case of matrix multiplication where each multiplication and addition can be subject to overflows. Thus

```
octave:1> x = randn(100,100);
octave:2> y0 = fixed(7,6,x*x);
octave:3> y1 = fixed(7,6,x)*fixed(7,6,x);
```

does not give equivalent operations for *y0* and *y1*. With all this additionally checking, you can not expect your code to run as fast when using the fixed point type.

When running under cygwin I get a dlopen error.

The build under cygwin is slightly different, in that most of the fixed point functions are compiled as a shared library that is linked to the main oct-file. This is to allow other oct-files to use the fixed-point type which is not possible under cygwin otherwise, since compilation under cygwin requires that all symbols are resolved at compile time.

If the shared libraries are not installed somewhere that can be found when running octave, then you will get an error like

```
octave:1> a = fixed(7,2,1)
error: dlopen: Win32 error 126
error: ‘fixed’ undefined near line 1 column 5
error: evaluating assignment expression near line 1, column 3
```

There are two files ‘`liboctave_fixed.dll`’ and ‘`liboctave_fixed.dll.a`’ that must be installed. Typically, these should be installed in the same directory that you can ‘`liboctave.dll`’ and ‘`liboctave.dll.a`’ respectively. If you use the same `--prefix` option to configure both octave and octave-forge then this should happen automatically.

2 Using the fixed-point type in C++ and oct-files

Octave supplies a matrix template library to create matrix and vector objects from basic types. All of the properties of these Octave classes will not be described here. However the base classes and the particularly of the classes created using the Octave matrix templates will be discussed.

There are two basic fixed point types: `FixedPoint` and `FixedPointComplex` representing the fixed point representation of real and complex numbers respectively. The Octave matrix templates are used to created the classes `FixedMatrix`, `FixedRowVector` and `FixedColumnVector` from the base class `FixedPoint`. Similar the complex fixed point types `FixedComplexMatrix`, `FixedComplexRowVector` and `FixedComplexColumnVectors` are constructed from the base class `FixedPointComplex`

This section discusses the definitions of the base classes, their extension with the Octave matrix templates, the upper level Octave classes and the use of all of these when writing oct-files.

2.1 The `FixedPoint` Base Class

2.1.1 `FixedPoint` Constructors

`FixedPoint::FixedPoint ()`
: Create a fixed point object with only a sign bit

`FixedPoint::FixedPoint (unsigned int is, unsigned int ds)`
: Create a fixed point object with `is` bits for the integer part and `ds` bits for the decimal part. The fixed point object will be initialized to be zero

`FixedPoint::FixedPoint (unsigned int is, unsigned int ds, FixedPoint &x)`
: Create a fixed point object with `is` bits for the integer part and `ds` bits for the decimal part, loaded with the fixed point value `x`. If `is + ds` is greater than `sizeof(int)*8 - 2`, the error handler is called.

`FixedPoint::FixedPoint (unsigned int is, unsigned int ds, const double x)`
: Create a fixed point object with `is` bits for the integer part and `ds` bits for the decimal part, loaded with the value `x`. If `is + ds` is greater than `sizeof(int)*8 - 2`, the error handler is called.

`FixedPoint::FixedPoint (unsigned int is, unsigned int ds, unsigned int i, unsigned int d)`
: Create a fixed point object with `is` bits for the integer part and `ds` bits for the decimal part, loading the integer part with `i` and the decimal part with `d`. It should be noted that `i` and `d` are both unsigned and are the strict representation of the bits of the fixed point value.

`FixedPoint::FixedPoint (const int x)`
: Create a fixed point object with the minimum number of bits for the integer part `is` needed to represent `x`. If `is` is greater than `sizeof(int)*8 - 2`, the error handler is called.

`FixedPoint::FixedPoint (const double x)`
: Create a fixed point object with the minimum number of bits for the integer part `is` needed to represent the integer part of `x`. If `is` is greater than `sizeof(int)*8 - 2`, the error handler is called.

`FixedPoint::FixedPoint (const FixedPoint &x)`
: Create a copy of the fixed point object `x`

2.1.2 `FixedPoint` Specific Methods

`double FixedPoint::fixedpoint()`
: Method to create a `double` from the current fixed point object

`double fixedpoint (const FixedPoint &x)`
: Create a `double` from the fixed point object `x`

`int FixedPoint::sign ()`
: Return `-1` for negative numbers, `1` for positive and `0` if the current fixed point object is zero.

`int sign (FixedPoint &x)`
: Return `-1` for negative numbers, `1` for positive and `0` if the fixed point object `x` is zero.

`char FixedPoint::signbit ()`
: Return the sign bit of the current fixed point number (`0` for positive number, `1` for negative number).

`char signbit (FixedPoint &x)`
: Return the sign bit of the fixed point number `x` (`0` for positive number, `1` for negative number).

`int FixedPoint::getintsize ()`
: Return the number of bit `is` used to represent the integer part of the current fixed point object.

`int getintsize (FixedPoint &x)`
: Return the number of bit `is` used to represent the integer part of the fixed point object `x`.

`int FixedPoint::getdecsize ()`
: Return the number of bit `ds` used to represent the decimal part of the current fixed point object.

`int getdecsize (FixedPoint &x)`
: Return the number of bit `ds` used to represent the decimal part of the fixed point object `x`.

`unsigned int FixedPoint::getnumber ()`
: Return the integer representation of the fixed point value of the current fixed point object.

`unsigned int getnumber (FixedPoint &x)`
: Return the integer representation of the fixed point value of the fixed point object `x`.

`FixedPoint FixedPoint::chintsize (const int n)`
: Return a fixed point object equivalent to the current fixed point number but with the number of bits representing the integer part `is` changed to `n`. If the result of this operation causes `is + ds` to be greater than `sizeof(int)*8 - 2`, the error handler is called.

`FixedPoint FixedPoint::chdecsize (const int n)`
: Return a fixed point object equivalent to the current fixed point number but with the number of bits representing the decimal part `ds` changed to `n`. If the result of this operation causes `is + ds` to be greater than `sizeof(int)*8 - 2`, the error handler is called.

`FixedPoint FixedPoint::incintsize (const int n)`
: Return a fixed point object equivalent to the current fixed point number but with the number of bits representing the integer part `is` increased by `n`. If `n` is negative, then `is` is decreased. If the result of this operation causes `is + ds` to be greater than `sizeof(int)*8 - 2`, the error handler is called.

`FixedPoint FixedPoint::incintsize ()`
: Return a fixed point object equivalent to the current fixed point number but with the number of bits representing the integer part `is` increased by 1. If the result of this operation causes `is + ds` to be greater than `sizeof(int)*8 - 2`, the error handler is called.

`FixedPoint FixedPoint::incdecsize (const int n)`
: Return a fixed point object equivalent to the current fixed point number but with the number of bits representing the decimal part `ds` increased by `n`. If `n` is negative, then `ds` is decreased. If the result of this operation causes `is + ds` to be greater than `sizeof(int)*8 - 2`, the error handler is called.

`FixedPoint FixedPoint::incdecsize ()`
: Return a fixed point object equivalent to the current fixed point number but with the number of bits representing the decimal part `ds` increased by 1. If the result of this operation causes `is + ds` to be greater than `sizeof(int)*8 - 2`, the error handler is called.

2.1.3 `FixedPoint` Operators

`FixedPoint operator +`
: Unary `+` of a fixed point object

`FixedPoint operator -`
: Unary `-` of a fixed point object

`FixedPoint operator !`
: Unary `!` operator of the fixedpoint object, with the the sign bit. This is not the same operator as `-` and is not the same operator as the octave `!` operator.

`FixedPoint operator ++`
: Unary increment operator (pre and postfix). Uses the smallest representable value to increment (ie 2^{-ds})

`FixedPoint operator --`
: Unary decrement operator (pre and postfix). Uses the smallest representable value to decrement (ie 2^{-ds})

`FixedPoint operator = (const FixedPoint &x)`
: Assignment operators. Copies fixed point object `x`

`FixedPoint operator += (const FixedPoint &x)`
`FixedPoint operator -= (const FixedPoint &x)`
`FixedPoint operator *= (const FixedPoint &x)`
`FixedPoint operator /= (const FixedPoint &x)`
: Assignment operators, working on both the input and output objects. The output object's fixed point representation is promoted such that the largest values of `is` and `ds` are taken from the input and output objects. If the result of this operation causes `is + ds` to be greater than `sizeof(int)*8 - 2`, the error handler is called.

`FixedPoint operator <<= (const int s)`
`FixedPoint operator << (const FixedPoint &x, const int s)`
: Perform a left-shift of a fixed point object. This is equivalent to a multiplication by a power of 2. The sign-bit is preserved. This differs from the `rshift` function discussed below in the `is` and `ds` are unchanged.

`FixedPoint operator >>= (const int s)`
`FixedPoint operator >> (const FixedPoint &x, const int s)`
: Perform a right-shift of the fixed point object. This is equivalent to a division by a power of 2. Note that the sign-bit is preserved. This differs from the `rshift` function discussed below in the `is` and `ds` are unchanged.

`FixedPoint operator + (const FixedPoint &x, const FixedPoint &y)`
`FixedPoint operator - (const FixedPoint &x, const FixedPoint &y)`
`FixedPoint operator * (const FixedPoint &x, const FixedPoint &y)`
`FixedPoint operator / (const FixedPoint &x, const FixedPoint &y)`
: Two argument operators. The output objects fixed point representation is promoted such that the largest values of `is` and `ds` are taken from the two arguments. If the result of this operation causes `is + ds` to be greater than `sizeof(int)*8 - 2`, the error handler is called.

`bool operator == (const FixedPoint &x, const FixedPoint &y)`
`bool operator != (const FixedPoint &x, const FixedPoint &y)`
`bool operator < (const FixedPoint &x, const FixedPoint &y)`
`bool operator <= (const FixedPoint &x, const FixedPoint &y)`
`bool operator > (const FixedPoint &x, const FixedPoint &y)`
`bool operator >= (const FixedPoint &x, const FixedPoint &y)`
: Fixed point comparison operators. The fixed point object `x` and `y` can have different representations (values of `is` and `ds`).

`std::istream &operator >> (std::istream &s, FixedPoint &x)`
: Read a fixed point object from `s` stream and store it into `x` keeping the fixed point representation in `x`. If the value read is not a fixed point object, the error handler is invoked.

`std::ostream &operator << (std::ostream &s, const FixedPoint &x)`
Send into the stream `s`, a formatted fixed point value `x`

2.1.4 `FixedPoint` Functions

`FixedPoint rshift (const FixedPoint &x, int s)`
Do a right shift of `s` bits of a fixed precision number (equivalent to a division by a power of 2). The representation of the fixed point object is adjusted to suit the new fixed point object (i.e. `ds++` and `is = is == 0 ? 0 : is-1`)

`FixedPoint lshift (const FixedPoint &x, int s)`
Do a left shift of `s` bits of a fixed precision number (equivalent to a multiplication by a power of 2). The representation of the fixed point object is adjusted to suit the new fixed point object (i.e. `is++` and `ds = ds == 0 ? 0 : ds-1`)

`FixedPoint abs (const FixedPoint &x)`
Returns the modulus of `x`

`FixedPoint cos (const FixedPoint &x)`
Returns the cosine of `x`

`FixedPoint cosh (const FixedPoint &x)`
Returns the hyperbolic cosine of `x`

`FixedPoint sin (const FixedPoint &x)`
Returns the sine of `x`

`FixedPoint sinh (const FixedPoint &x)`
Returns the hyperbolic sine of `x`

`FixedPoint tan (const FixedPoint &x)`
Returns the tangent of `x`

`FixedPoint tanh (const FixedPoint &x)`
Returns the hyperbolic tangent of `x`

`FixedPoint sqrt (const FixedPoint &x)`
Returns the square root of `x`

`FixedPoint pow (const FixedPoint &x, int y)`
`FixedPoint pow (const FixedPoint &x, double y)`
`FixedPoint pow (const FixedPoint &x, const FixedPoint &y)`
Returns the `x` raised to the power `y`

`FixedPoint exp (const FixedPoint &x)`
Returns the exponential of `x`

`FixedPoint log (const FixedPoint &x)`
Returns the logarithm of `x`

`FixedPoint log10 (const FixedPoint &x)`
Returns the base 10 logarithm of `x`

`FixedPoint atan2 (const FixedPoint &y, const FixedPoint &x)`
Returns the arc tangent of `x` and `y`

`FixedPoint floor (const FixedPoint &x)`
: Returns the rounded value of `x` downwards to the nearest integer

`FixedPoint ceil (const FixedPoint &x)`
: Returns the rounded value of `x` upwards to the nearest integer

`FixedPoint rint (const FixedPoint &x)`
: Returns the rounded value of `x` to the nearest integer

`FixedPoint round (const FixedPoint &x)`
: Returns the rounded value of `x` to the nearest integer. The difference with `rint` is that `0.5` is rounded to `1` and not `0`. This conforms to the behavior of the octave `round` function.

`std::string getbitstring (const FixedPoint &x)`
: Return a string containing the bits of `x`

2.2 The `FixedPointComplex` Base Class

The `FixedPointComplex` class is derived using the C++ compilers inbuilt `complex` class and is instantiated as `std::complex<FixedPoint>`. Therefore the exact behavior of the complex fixed point type is determined by the complex class used by the C++ compiler. The user is advised to understand their C++ compilers implementation of the complex functions that they use, and particularly the effects that they will have on the precision of fixed point operations.

2.2.1 `FixedPointComplex` Constructors

`FixedPointComplex::FixedPointComplex ()`
: Create a complex fixed point object with only a sign bit

`FixedPointComplex::FixedPointComplex (unsigned int is, unsigned int ds)`
: Create a complex fixed point object with `is` bits for the integer part and `ds` bits for the decimal part. The fixed point object will be initialized to zero.

`FixedPointComplex::FixedPointComplex (unsigned int is, unsigned int ds, FixedPoint &x)`
: Create a complex fixed point object with `is` bits for the integer part and `ds` bits for the decimal part of both real and imaginary parts, loaded with the fixed point value `x` in the real part and zero in the imaginary. If `is + ds` is greater than `sizeof(int)*8 - 2`, the error handler is called.

`FixedPointComplex::FixedPointComplex (unsigned int is, unsigned int ds, FixedPoint &r, FixedPoint &i)`
: Create a complex fixed point object with `is` bits for the integer part and `ds` bits for the decimal part of both real and imaginary parts, loaded with the fixed point value `r` in the real part and `i` the imaginary part. If `is + ds` is greater than `sizeof(int)*8 - 2`, the error handler is called.

`FixedPointComplex::FixedPointComplex (unsigned int is, unsigned int ds, FixedPointComplex &x)`
: Create a complex fixed point object with `is` bits for the integer part and `ds` bits for the decimal part of both real and imaginary parts, loaded with the complex

fixed point value x. If is + ds is greater than sizeof(int)*8 - 2, the error handler is called.

`FixedPointComplex::FixedPointComplex (unsigned int is, unsigned int ds, const double x)`

Create a complex fixed point object with is bits for the integer part and ds bits for the decimal part of both real and imaginary parts, loaded with the fixed point value x in the real part and zero in the imaginary. If is + ds is greater than sizeof(int)*8 - 2, the error handler is called.

`FixedPointComplex::FixedPointComplex (unsigned int is, unsigned int ds, const double r, const double i)`

Create a complex fixed point object with is bits for the integer part and ds bits for the decimal part of both real and imaginary parts, loaded with the fixed point value r in the real part and i in the imaginary part. If is + ds is greater than sizeof(int)*8 - 2, the error handler is called.

`FixedPointComplex::FixedPointComplex (unsigned int is, unsigned int ds, const Complex c)`

Create a complex fixed point object with is bits for the integer part and ds bits for the decimal part of both real and imaginary parts, loaded with the fixed point value c in the real and imaginary parts. If is + ds is greater than sizeof(int)*8 - 2, the error handler is called.

`FixedPointComplex::FixedPointComplex (unsigned int is, unsigned int ds, const Complex a, const Complex b)`

Create a complex fixed point object with is bits for the integer part and ds bits for the decimal part of both real and imaginary parts, loaded with the fixed point value a in the integer parts of the of the value and b in the decimal part. It should be noted that a and b are both unsigned and are the strict representation of the bits of the fixed point value and considered as integers. If is + ds is greater than sizeof(int)*8 - 2, the error handler is called.

`FixedPointComplex::FixedPointComplex (const Complex& is, const Complex& ds)`

Create a complex fixed point object with is bits for the integer part and ds bits for the decimal part of both real and imaginary parts, loaded with zero in the real and imaginary parts. The real part of is is used for the real part of the complex fixed point object, while the imaginary part of is is used for the imaginary part. If either the sum of the real or imaginary parts of is + ds is greater than sizeof(int)*8 - 2, the error handler is called.

`FixedPointComplex::FixedPointComplex (const Complex& is, const Complex& ds, const Complex& c)`

Create a complex fixed point object with is bits for the integer part and ds bits for the decimal part of both real and imaginary parts, loaded with the complex fixed point value c in the real and imaginary parts. The real part of is is used for the real part of the complex fixed point object, while the imaginary part of is is used for the imaginary part. If either the sum of the real or imaginary parts of is + ds is greater than sizeof(int)*8 - 2, the error handler is called.

`FixedPointComplex::FixedPointComplex (const Complex& is, const Complex& ds, const FixedPointComplex& c)`

Create a complex fixed point object with `is` bits for the integer part and `ds` bits for the decimal part of both real and imaginary parts, loaded with the complex fixed point value `c` in the real and imaginary parts. The real part of `is` is used for the real part of the complex fixed point object, while the imaginary part of `is` is used for the imaginary part. If either the sum of the real or imaginary parts of `is + ds` is greater than `sizeof(int)*8 - 2`, the error handler is called.

`FixedPointComplex::FixedPointComplex (const Complex& is, const Complex& ds, const Complex& a, const Complex& b)`

Create a complex fixed point object with `is` bits for the integer part and `ds` bits for the decimal part of both real and imaginary parts, loaded with the fixed point value `a` in the integer parts of the of the value and `b` in the decimal part. It should be noted that `a` and `b` are both unsigned and are the strict representation of the bits of the fixed point value and considered as integers. If either the sum of the real or imaginary parts of `is + ds` is greater than `sizeof(int)*8 - 2`, the error handler is called.

`FixedPointComplex::FixedPointComplex (const std::complex<FixedPoint> &c)`

Create a complex fixed point object with the minimum number of bits for the integer part `is` needed to represent the real and imaginary integer parts of `x`. If `is` is greater than `sizeof(int)*8 - 2`, the error handler is called. The real and imaginary parts are treated separately.

`FixedPointComplex::FixedPointComplex (const FixedPointComplex &x)`

Create a copy of the complex fixed point object `x`

`FixedPointComplex::FixedPointComplex (const FixedPoint &x)`

Create a copy of the fixed point object `x`, into a complex fixed point object leaving the imaginary part at zero.

`FixedPointComplex::FixedPointComplex (const FixedPoint &r, const FixedPoint &i)`

Create a copy of the fixed point objects `r` and `i`, into a the real and imaginary parts of a complex fixed point object respectively

`FixedPointComplex::FixedPointComplex (const int x)`

Create a fixed point object with the minimum number of bits for the integer part `is` needed to represent `x`. If `is` is greater than `sizeof(int)*8 - 2`, the error handler is called.

`FixedPointComplex::FixedPointComplex (const double x)`

Create a fixed point object with the minimum number of bits for the integer part `is` needed to represent the integer part of `x`. If `is` is greater than `sizeof(int)*8 - 2`, the error handler is called.

`FixedPointComplex (const Complex& is, const Complex& ds, const double& d)`

Create a complex fixed point object with `is` bits for the integer part and `ds` bits for the decimal part of both real and imaginary parts, loaded with the double value `d` in the real and zero in the imaginary parts. The real part of `is` is used

for the real part of the complex fixed point object, while the imaginary part of `is` is used for the imaginary part. If either the sum of the real or imaginary parts of `is + ds` is greater than `sizeof(int)*8 - 2`, the error handler is called.

2.2.2 `FixedPointComplex` Specific Methods

Complex **FixedPointComplex::fixedpoint()**
: Method to create a `Complex` from the current fixed point object

Complex **fixedpoint (const FixedPointComplex &x)**
: Create a `Complex` from the fixed point object `x`

Complex **FixedPointComplex::sign ()**
: Return `-1` for negative numbers, `1` for positive and `0` if the current fixed point object is zero. Real and imaginary parts treated separately and returned in the real and imaginary parts of the return object.

Complex **sign (FixedPointComplex &x)**
: Return `-1` for negative numbers, `1` for positive and `0` if the fixed point object `x` is zero. Real and imaginary parts treated separately and returned in the real and imaginary parts of the return object.

Complex **FixedPointComplex::getintsize ()**
: Return the number of bit `is` used to represent the integer part of the current fixed point object. Real and imaginary parts treated separately and returned in the real and imaginary parts of the return object.

Complex **getintsize (FixedPointComplex &x)**
: Return the number of bit `is` used to represent the integer part of the fixed point object `x`. Real and imaginary parts treated separately and returned in the real and imaginary parts of the return object.

Complex **FixedPointComplex::getdecsize ()**
: Return the number of bit `ds` used to represent the decimal part of the current fixed point object. Real and imaginary parts treated separately and returned in the real and imaginary parts of the return object.

Complex **getdecsize (FixedPointComplex &x)**
: Return the number of bit `ds` used to represent the decimal part of the fixed point object `x`. Real and imaginary parts treated separately and returned in the real and imaginary parts of the return object.

Complex **FixedPointComplex::getnumber ()**
: Return the integer representation of the fixed point value of the current fixed point object. Real and imaginary parts treated separately and returned in the real and imaginary parts of the return object.

Complex **getnumber (FixedPointComplex &x)**
: Return the integer representation of the fixed point value of the fixed point object `x`. Real and imaginary parts treated separately and returned in the real and imaginary parts of the return object.

`FixedPointComplex` `FixedPointComplex::chintsize (const Complex n)`
: Return a fixed point object equivalent to the current fixed point number but with the number of bits representing the integer part `is` changed to `n`. If the result of this operation causes `is + ds` to be greater than `sizeof(int)*8 - 2`, the error handler is called. Real and imaginary parts treated separately and returned in the real and imaginary parts of the return object.

`FixedPointComplex` `FixedPointComplex::chdecsize (const Complex n)`
: Return a fixed point object equivalent to the current fixed point number but with the number of bits representing the decimal part `ds` changed to `n`. If the result of this operation causes `is + ds` to be greater than `sizeof(int)*8 - 2`, the error handler is called. Real and imaginary parts treated separately and returned in the real and imaginary parts of the return object.

`FixedPointComplex` `FixedPointComplex::incintsize (const Complex n)`
: Return a fixed point object equivalent to the current fixed point number but with the number of bits representing the integer part `is` increased by `n`. If `n` is negative, then `is` is decreased. If the result of this operation causes `is + ds` to be greater than `sizeof(int)*8 - 2`, the error handler is called. Real and imaginary parts treated separately and returned in the real and imaginary parts of the return object.

`FixedPointComplex` `FixedPointComplex::incintsize ()`
: Return a fixed point object equivalent to the current fixed point number but with the number of bits representing the integer part `is` increased by 1. If the result of this operation causes `is + ds` to be greater than `sizeof(int)*8 - 2`, the error handler is called. Real and imaginary parts treated separately and returned in the real and imaginary parts of the return object.

`FixedPointComplex` `FixedPointComplex::incdecsize (const Complex n)`
: Return a fixed point object equivalent to the current fixed point number but with the number of bits representing the decimal part `ds` increased by `n`. If `n` is negative, then `ds` is decreased. If the result of this operation causes `is + ds` to be greater than `sizeof(int)*8 - 2`, the error handler is called. Real and imaginary parts treated separately and returned in the real and imaginary parts of the return object.

`FixedPointComplex` `FixedPointComplex::incdecsize ()`
: Return a fixed point object equivalent to the current fixed point number but with the number of bits representing the decimal part `ds` increased by 1. If the result of this operation causes `is + ds` to be greater than `sizeof(int)*8 - 2`, the error handler is called. Real and imaginary parts treated separately and returned in the real and imaginary parts of the return object.

2.2.3 `FixedPointComplex` Operators

`FixedPointComplex` `operator +`
: Unary `+` of a complex fixed point object

`FixedPointComplex` `operator -`
: Unary `-` of a complex fixed point object

`FixedPointComplex operator = (const FixedPointComplex &x)`

Assignment operators. Copies complex fixed point object `x`

`FixedPointComplex operator += (const FixedPointComplex &x)`
`FixedPointComplex operator -= (const FixedPointComplex &x)`
`FixedPointComplex operator *= (const FixedPointComplex &x)`
`FixedPointComplex operator /= (const FixedPointComplex &x)`

Assignment operators, working on both the input and output objects. The output object's fixed point representation is promoted such that the largest values of `is` and `ds` are taken from the input and output objects. If the result of this operation causes `is + ds` to be greater than `sizeof(int)*8 - 2`, the error handler is called.

`FixedPointComplex operator + (const FixedPointComplex &x, const FixedPointComplex &y)`
`FixedPointComplex operator - (const FixedPointComplex &x, const FixedPointComplex &y)`
`FixedPointComplex operator * (const FixedPointComplex &x, const FixedPointComplex &y)`
`FixedPointComplex operator / (const FixedPointComplex &x, const FixedPointComplex &y)`

Two argument operators. The output objects complex fixed point representation is promoted such that the largest values of `is` and `ds` are taken from the two arguments. If the result of this operation causes `is + ds` to be greater than `sizeof(int)*8 - 2`, the error handler is called.

`bool operator == (const FixedPointComplex &x, const FixedPointComplex &y)`
`bool operator != (const FixedPointComplex &x, const FixedPointComplex &y)`

Complex fixed point comparison operators. The complex fixed point object `x` and `y` can have different representations (values of `is` and `ds`).

`std::istream &operator >> (std::istream &s, FixedPointComplex &x)`

Read a fixed point object from `s` stream and store it into `x` keeping the fixed point representation in `x`. If the value read is not a fixed point object, the error handler is invoked.

`std::ostream &operator << (std::ostream &s, const FixedPointComplex &x)`

Send into the stream `s`, a formatted fixed point value `x`

2.2.4 `FixedPointComplex` Functions

`FixedPoint abs (const FixedPointComplex &x)`

Returns the modulus of `x`

`FixedPoint norm (const FixedPointComplex &x)`

Returns the squared magnitude of `x`

`FixedPoint arg (const FixedPointComplex &x)`

Returns the arc-tangent of `x`

`FixedPointComplex std::polar (const FixedPoint &r, const FixedPoint &p)`

Convert from polar fixed point to a complex fixed point object

`FixedPoint real (const FixedPointComplex &x)`
: Returns the real part of `x`

`FixedPoint imag (const FixedPointComplex &x)`
: Returns the imaginary part of `x`

`FixedPointComplex conj (const FixedPointComplex &x)`
: Returns the conjugate of `x`

`FixedPointComplex cos (const FixedPointComplex &x)`
: Returns the transcendental cosine of `x`

`FixedPointComplex cosh (const FixedPointComplex &x)`
: Returns the transcendental hyperbolic cosine of `x`

`FixedPointComplex sin (const FixedPointComplex &x)`
: Returns the transcendental sine of `x`

`FixedPointComplex sinh (const FixedPointComplex &x)`
: Returns the transcendental hyperbolic sine of `x`

`FixedPointComplex tan (const FixedPointComplex &x)`
: Returns the transcendental tangent of `x`

`FixedPointComplex tanh (const FixedPointComplex &x)`
: Returns the transcendental hyperbolic tangent of `x`

`FixedPointComplex sqrt (const FixedPointComplex &x)`
: Returns the square root of `x`

`FixedPointComplex pow (const FixedPointComplex &x, int y)`
`FixedPointComplex pow (const FixedPointComplex &x, const FixedPoint &y)`
`FixedPointComplex pow (const FixedPointComplex &x, const FixedPointComplex &y)`
`FixedPointComplex pow (const FixedPoint &x, const FixedPointComplex &y)`
: Returns the `x` raised to the power `y`. Be careful of precision errors associated with the implementation of this function as a complex type

`FixedPointComplex exp (const FixedPointComplex &x)`
: Returns the exponential of `x`. Be careful of precision errors associated with the implementation of this function as a complex type

`FixedPointComplex log (const FixedPointComplex &x)`
: Returns the transcendental logarithm of `x`. Be careful of precision errors associated with the implementation of this function as a complex type

`FixedPointComplex log10 (const FixedPointComplex &x)`
: Returns the transcendental base 10 logarithm of `x`. Be careful of precision errors associated with the implementation of this function as a complex type

`FixedPointComplex floor (const FixedPointComplex &x)`
: Returns the rounded value of `x` downwards to the nearest integer, treating the real and imaginary parts separately

`FixedPointComplex ceil (const FixedPointComplex &x)`
: Returns the rounded value of `x` upwards to the nearest integer, treating the real and imaginary parts separately

`FixedPointComplex rint (const FixedPointComplex &x)`
: Returns the rounded value of `x` to the nearest integer, treating the real and imaginary parts separately

`FixedPointComplex round (const FixedPointComplex &x)`
: Returns the rounded value of `x` to the nearest integer. The difference with `rint` is that `0.5` is rounded to `1` and not `0`. This conforms to the behavior of the octave `round` function. Treats the real and imaginary parts separately

2.3 The Derived Classes using the Octave Template Classes

It is not the purpose of this section to discuss the use of the Octave template classes, but rather only the additions to the fixed point classes that are based on these. For instance the basic constructors and operations that are available in the normal floating point Octave classes are available within the fixed point classes.

The notable exceptions are operations involving matrix decompositions, including inversion, left division, etc. The reason for this is that the precision of these operators and functions will be highly implementation dependent. As additional these operators and functions are used rarely with a fixed point type, there is no point in implementing these operators and function within the fixed point classes.

In addition the the functions and operators previously described for the `FixedPoint` and the `FixedPointComplex` types are all available, in the same from as previously. So these functions are not documented below. Only the constructors and methods that vary from the previously described versions are described.

To fully understand these classes, the user is advised to examine the header files '`fixedMatrix.h`', '`fixedRowVector.h`' and '`fixedColVector.h`' for the real fixed point objects and '`fixedCMatrix.h`', '`fixedCRowVector.h`' and '`fixedCColVector.h`' for the complex fixed point objects. In addition the files '`MArray.h`' and '`MArray2.h`' from Octave should also be examined.

2.3.1 `FixedMatrix` class

`FixedMatrix::FixedMatrix (const MArray2<int> &is, const MArray2<int> &ds)`
`FixedMatrix::FixedMatrix (const Matrix &is, const Matrix &ds)`
: Create a fixed point matrix with the number of bits in the integer part of each element represented by `is` and the number in the decimal part by `ds`. The fixed point elements themselves will be initialized to zero.

FixedMatrix::FixedMatrix (unsigned int is, unsigned int ds, const FixedMatrix& a)
FixedMatrix::FixedMatrix (const MArray2<int> &is, const MArray2<int> &ds, const FixedMatrix& a)
FixedMatrix::FixedMatrix (const Matrix &is, const Matrix &ds, const FixedMatrix& a)

Create a fixed point matrix with the number of bits in the integer part of each element represented by is and the number in the decimal part by ds. The fixed point elements themselves will be initialized to the fixed point matrix a

FixedMatrix::FixedMatrix (unsigned int is, unsigned int ds, const Matrix& a)
FixedMatrix::FixedMatrix (const MArray2<int> &is, const MArray2<int> &ds, const Matrix& a)
FixedMatrix::FixedMatrix (const Matrix &is, const Matrix &ds, const Matrix& a)

Create a fixed point matrix with the number of bits in the integer part of each element represented by is and the number in the decimal part by ds. The fixed point elements themselves will be initialized to the matrix a

FixedMatrix::FixedMatrix (unsigned int is, unsigned int ds, const Matrix& a, const Matrix& b)
FixedMatrix::FixedMatrix (const MArray2<int> &is, const MArray2<int> &ds, const Matrix& a, const Matrix& b)
FixedMatrix::FixedMatrix (const Matrix &is, const Matrix &ds, const Matrix& a, const Matrix& b)

Create a fixed point matrix with the number of bits in the integer part of each element represented by is and the number in the decimal part by ds. The fixed point elements themselves will have the integer parts loaded by the matrix a and the decimal part by the matrix b. It should be noted that a and b are both considered as unsigned integers and are the strict representation of the bits of the fixed point value.

Matrix FixedMatrix::fixedpoint ()

Method to create a Matrix from a fixed point matrix x

Matrix fixedpoint (const FixedMatrix &x)

Create a Matrix from a fixed point matrix x

Matrix FixedMatrix::sign ()

Return -1 for negative numbers, 1 for positive and 0 if the fixed point element is zero, for every element of the current fixed point object.

Matrix sign (const FixedMatrix &x)

Return -1 for negative numbers, 1 for positive and 0 if zero, for every element of the fixed point object x.

Matrix FixedMatrix::signbit ()

Return the sign bit for every element of the current fixed point matrix (0 for positive number, 1 for negative number).

Matrix signbit (const FixedMatrix &x)

Return the sign bit for every element of the fixed point matrix x (0 for positive number, 1 for negative number).

`Matrix FixedMatrix::getintsize ()`
: Return the number of bit `is` used to represent the integer part of each element of the current fixed point object.

`Matrix getintsize (const FixedMatrix &x)`
: Return the number of bit `is` used to represent the integer part of each element of the fixed point object `x`.

`Matrix FixedMatrix::getdecsize ()`
: Return the number of bit `ds` used to represent the decimal part of each element of the current fixed point object.

`Matrix getdecsize (const FixedMatrix &x)`
: Return the number of bit `ds` used to represent the decimal part of each element of the fixed point object `x`.

`Matrix FixedMatrix::getnumber ()`
: Return the integer representation of the fixed point value of the each eleement of the current fixed point object.

`Matrix getnumber (const FixedMatrix &x)`
: Return the integer representation of the fixed point value of the each eleement of the fixed point object `x`.

`FixedMatrix FixedMatrix::chintsize (const Matrix &n)`
`FixedMatrix FixedMatrix::chintsize (const double n)`
: Return a fixed point object equivalent to the current fixed point object but with the number of bits representing the integer part `is` of every element changed to `n`. If the result of this operation causes any element of `is + ds` to be greater than `sizeof(int)*8 - 2`, the error handler is called.

`FixedMatrix FixedMatrix::chdecsize (const double n)`
`FixedMatrix FixedMatrix::chdecsize (const Matrix &n)`
: Return a fixed point object equivalent to the current fixed point object but with the number of bits representing the decimal part `ds` of every element changed to `n`. If the result of this operation causes any element of `is + ds` to be greater than `sizeof(int)*8 - 2`, the error handler is called.

`FixedMatrix FixedMatrix::incintsize (const double n)`
`FixedMatrix FixedMatrix::incintsize (const Matrix &n)`
: Return a fixed point object equivalent to the current fixed point number but with the number of bits representing the integer part `is` increased by `n`. If `n` is negative, then `is` is decreased. If the result of this operation causes `is + ds` to be greater than `sizeof(int)*8 - 2`, the error handler is called.

`FixedMatrix FixedMatrix::incintsize ()`
: Return a fixed point object equivalent to the current fixed point number but with the number of bits representing the integer part `is` increased by 1. If the result of this operation causes `is + ds` to be greater than `sizeof(int)*8 - 2`, the error handler is called.

`FixedMatrix FixedMatrix::incdecsize (const double n)`
`FixedMatrix FixedMatrix::incdecsize (const Matrix &n)`

Return a fixed point object equivalent to the current fixed point number but with the number of bits representing the decimal part `ds` increased by `n`. If `n` is negative, then `ds` is decreased. If the result of this operation causes `is + ds` to be greater than `sizeof(int)*8 - 2`, the error handler is called.

`FixedMatrix FixedMatrix::incdecsize ()`

Return a fixed point object equivalent to the current fixed point number but with the number of bits representing the decimal part `ds` increased by 1. If the result of this operation causes `is + ds` to be greater than `sizeof(int)*8 - 2`, the error handler is called.

2.3.2 `FixedRowVector` class

`FixedRowVector::FixedRowVector (const MArray<int> &is, const MArray<int> &ds)`
`FixedRowVector::FixedRowVector (const RowVector &is, const RowVector &ds)`

Create a fixed point row vector with the number of bits in the integer part of each element represented by `is` and the number in the decimal part by `ds`. The fixed point elements themselves will be initialized to zero.

`FixedRowVector::FixedRowVector (unsigned int is, unsigned int ds, const FixedRowVector& a)`
`FixedRowVector::FixedRowVector (const MArray<int> &is, const MArray<int> &ds, const FixedRowVector& a)`
`FixedRowVector::FixedRowVector (const RowVector &is, const RowVector &ds, const FixedRowVector& a)`

Create a fixed point row vector with the number of bits in the integer part of each element represented by `is` and the number in the decimal part by `ds`. The fixed point elements themselves will be initialized to the fixed point row rector `a`

`FixedRowVector::FixedRowVector (unsigned int is, unsigned int ds, const RowVector& a)`
`FixedRowVector::FixedRowVector (const MArray<int> &is, const MArray<int> &ds, const RowVector& a)`
`FixedRowVector::FixedRowVector (const RowVector &is, const RowVector &ds, const RowVector& a)`

Create a fixed point row vector with the number of bits in the integer part of each element represented by `is` and the number in the decimal part by `ds`. The fixed point elements themselves will be initialized to the row vector `a`

`FixedRowVector::FixedRowVector (unsigned int is, unsigned int ds, const RowVector& a, const RowVector& b)`
`FixedRowVector::FixedRowVector (const MArray<int> &is, const MArray<int> &ds, const RowVector& a, const RowVector& b)`
`FixedRowVector::FixedRowVector (const RowVector &is, const RowVector &ds, const RowVector& a, const RowVector& b)`

Create a fixed point row vector with the number of bits in the integer part of each element represented by `is` and the number in the decimal part by `ds`. The

fixed point elements themselves will have the integer parts loaded by the row vector `a` and the decimal part by the row vector `b`. It should be noted that `a` and `b` are both considered as unsigned integers and are the strict representation of the bits of the fixed point value.

`RowVector FixedRowVector::fixedpoint ()`
: Method to create a `RowVector` from a fixed point row vector `x`

`RowVector fixedpoint (const FixedRowVector &x)`
: Create a `RowVector` from a fixed point row vector `x`

`RowVector FixedRowVector::sign ()`
: Return `-1` for negative numbers, `1` for positive and `0` if the fixed point element is zero, for every element of the current fixed point object.

`RowVector sign (const FixedRowVector &x)`
: Return `-1` for negative numbers, `1` for positive and `0` if zero, for every element of the fixed point object `x`.

`RowVector FixedRowVector::signbit ()`
: Return the sign bit for every element of the current fixed point row vector (`0` for positive number, `1` for negative number).

`RowVector signbit (const FixedRowVector &x)`
: Return the sign bit for every element of the fixed point row vector `x` (`0` for positive number, `1` for negative number).

`RowVector FixedRowVector::getintsize ()`
: Return the number of bit `is` used to represent the integer part of each element of the current fixed point object.

`RowVector getintsize (const FixedRowVector &x)`
: Return the number of bit `is` used to represent the integer part of each element of the fixed point object `x`.

`RowVector FixedRowVector::getdecsize ()`
: Return the number of bit `ds` used to represent the decimal part of each element of the current fixed point object.

`RowVector getdecsize (const FixedRowVector &x)`
: Return the number of bit `ds` used to represent the decimal part of each element of the fixed point object `x`.

`RowVector FixedRowVector::getnumber ()`
: Return the integer representation of the fixed point value of the each eleement of the current fixed point object.

`RowVector getnumber (const FixedRowVector &x)`
: Return the integer representation of the fixed point value of the each eleement of the fixed point object `x`.

`FixedRowVector FixedRowVector::chintsize (const RowVector n)`
`FixedRowVector FixedRowVector::chintsize (const double n)`
: Return a fixed point object equivalent to the current fixed point object but with the number of bits representing the integer part `is` of every element changed

to n. If the result of this operation causes any element of is + ds to be greater than sizeof(int)*8 - 2, the error handler is called.

FixedRowVector FixedRowVector::chdecsize (const double n)
FixedRowVector FixedRowVector::chdecsize (const RowVector n)

Return a fixed point object equivalent to the current fixed point object but with the number of bits representing the decimal part ds of every element changed to n. If the result of this operation causes any element of is + ds to be greater than sizeof(int)*8 - 2, the error handler is called.

FixedRowVector FixedRowVector::incintsize (const double n)
FixedRowVector FixedRowVector::incintsize (const RowVector &n)

Return a fixed point object equivalent to the current fixed point number but with the number of bits representing the integer part is increased by n. If n is negative, then is is decreased. If the result of this operation causes is + ds to be greater than sizeof(int)*8 - 2, the error handler is called.

FixedRowVector FixedRowVector::incintsize ()

Return a fixed point object equivalent to the current fixed point number but with the number of bits representing the integer part is increased by 1. If the result of this operation causes is + ds to be greater than sizeof(int)*8 - 2, the error handler is called.

FixedRowVector FixedRowVector::incdecsize (const double n)
FixedRowVector FixedRowVector::incdecsize (const RowVector &n)

Return a fixed point object equivalent to the current fixed point number but with the number of bits representing the decimal part ds increased by n. If n is negative, then ds is decreased. If the result of this operation causes is + ds to be greater than sizeof(int)*8 - 2, the error handler is called.

FixedRowVector FixedRowVector::incdecsize ()

Return a fixed point object equivalent to the current fixed point number but with the number of bits representing the decimal part ds increased by 1. If the result of this operation causes is + ds to be greater than sizeof(int)*8 - 2, the error handler is called.

2.3.3 FixedColumnVector class

FixedColumnVector::FixedColumnVector (const MArray<int> &is, const MArray<int> &ds)
FixedColumnVector::FixedColumnVector (const ColumnVector &is, const ColumnVector &ds)

Create a fixed point row vector with the number of bits in the integer part of each element represented by is and the number in the decimal part by ds. The fixed point elements themselves will be initialized to zero.

FixedColumnVector::FixedColumnVector (unsigned int is, unsigned int ds, const FixedColumnVector& a)
FixedColumnVector::FixedColumnVector (const MArray<int> &is, const MArray<int> &ds, const FixedColumnVector& a)
FixedColumnVector::FixedColumnVector (const ColumnVector &is, const ColumnVector &ds, const FixedColumnVector& a)

Create a fixed point row vector with the number of bits in the integer part of each element represented by is and the number in the decimal part by ds. The fixed point elements themselves will be initialized to the fixed point column vector a

FixedColumnVector::FixedColumnVector (unsigned int is, unsigned int ds, const ColumnVector& a)
FixedColumnVector::FixedColumnVector (const MArray<int> &is, const MArray<int> &ds, const ColumnVector& a)
FixedColumnVector::FixedColumnVector (const ColumnVector &is, const ColumnVector &ds, const ColumnVector& a)

Create a fixed point row vector with the number of bits in the integer part of each element represented by is and the number in the decimal part by ds. The fixed point elements themselves will be initialized to the column vector a

FixedColumnVector::FixedColumnVector (unsigned int is, unsigned int ds, const ColumnVector& a, const ColumnVector& b)
FixedColumnVector::FixedColumnVector (const MArray<int> &is, const MArray<int> &ds, const ColumnVector& a, const ColumnVector& b)
FixedColumnVector::FixedColumnVector (const ColumnVector &is, const ColumnVector &ds, const ColumnVector& a, const ColumnVector& b)

Create a fixed point row vector with the number of bits in the integer part of each element represented by is and the number in the decimal part by ds. The fixed point elements themselves will have the integer parts loaded by the column vector a and the decimal part by the column vector b. It should be noted that a and b are both considered as unsigned integers and are the strict representation of the bits of the fixed point value.

ColumnVector FixedColumnVector::fixedpoint ()

Method to create a ColumnVector from a fixed point column vector x

ColumnVector fixedpoint (const FixedColumnVector &x)

Create a ColumnVector from a fixed point column vector x

ColumnVector FixedColumnVector::sign ()

Return -1 for negative numbers, 1 for positive and 0 if the fixed point element is zero, for every element of the current fixed point object.

ColumnVector sign (const FixedColumnVector &x)

Return -1 for negative numbers, 1 for positive and 0 if zero, for every element of the fixed point object x.

ColumnVector FixedColumnVector::signbit ()

Return the sign bit for every element of the current fixed point column vector (0 for positive number, 1 for negative number).

`ColumnVector signbit (const FixedColumnVector &x)`
: Return the sign bit for every element of the fixed point column vector `x` (`0` for positive number, `1` for negative number).

`ColumnVector FixedColumnVector::getintsize ()`
: Return the number of bit `is` used to represent the integer part of each element of the current fixed point object.

`ColumnVector getintsize (const FixedColumnVector &x)`
: Return the number of bit `is` used to represent the integer part of each element of the fixed point object `x`.

`ColumnVector FixedColumnVector::getdecsize ()`
: Return the number of bit `ds` used to represent the decimal part of each element of the current fixed point object.

`ColumnVector getdecsize (const FixedColumnVector &x)`
: Return the number of bit `ds` used to represent the decimal part of each element of the fixed point object `x`.

`ColumnVector FixedColumnVector::getnumber ()`
: Return the integer representation of the fixed point value of the each eleement of the current fixed point object.

`ColumnVector getnumber (const FixedColumnVector &x)`
: Return the integer representation of the fixed point value of the each eleement of the fixed point object `x`.

`FixedColumnVector FixedColumnVector::chintsize (const ColumnVector n)`
`FixedColumnVector FixedColumnVector::chintsize (const double n)`
: Return a fixed point object equivalent to the current fixed point object but with the number of bits representing the integer part `is` of every element changed to `n`. If the result of this operation causes any element of `is + ds` to be greater than `sizeof(int)*8 - 2`, the error handler is called.

`FixedColumnVector FixedColumnVector::chdecsize (const double n)`
`FixedColumnVector FixedColumnVector::chdecsize (const ColumnVector n)`
: Return a fixed point object equivalent to the current fixed point object but with the number of bits representing the decimal part `ds` of every element changed to `n`. If the result of this operation causes any element of `is + ds` to be greater than `sizeof(int)*8 - 2`, the error handler is called.

`FixedColumnVector FixedColumnVector::incintsize (const double n)`
`FixedColumnVector FixedColumnVector::incintsize (const ColumnVector &n)`
: Return a fixed point object equivalent to the current fixed point number but with the number of bits representing the integer part `is` increased by `n`. If `n` is negative, then `is` is decreased. If the result of this operation causes `is + ds` to be greater than `sizeof(int)*8 - 2`, the error handler is called.

`FixedColumnVector FixedColumnVector::incintsize ()`
: Return a fixed point object equivalent to the current fixed point number but with the number of bits representing the integer part `is` increased by 1. If the

result of this operation causes is + ds to be greater than sizeof(int)*8 - 2, the error handler is called.

FixedColumnVector FixedColumnVector::incdecsize (const double n)
FixedColumnVector FixedColumnVector::incdecsize (const ColumnVector &n)

Return a fixed point object equivalent to the current fixed point number but with the number of bits representing the decimal part ds increased by n. If n is negative, then ds is decreased. If the result of this operation causes is + ds to be greater than sizeof(int)*8 - 2, the error handler is called.

FixedColumnVector FixedColumnVector::incdecsize ()

Return a fixed point object equivalent to the current fixed point number but with the number of bits representing the decimal part ds increased by 1. If the result of this operation causes is + ds to be greater than sizeof(int)*8 - 2, the error handler is called.

2.3.4 FixedComplexMatrix class

FixedComplexMatrix::FixedComplexMatrix (const MArray2<int> &is, const MArray2<int> &ds)
FixedComplexMatrix::FixedComplexMatrix (const Matrix &is, const Matrix &ds)

Create a complex fixed point matrix with the number of bits in the integer part of the real and imaginary part of each element represented by is and the number in the decimal part by ds. The fixed point elements themselves will be initialized to zero.

FixedComplexMatrix::FixedComplexMatrix (const ComplexMatrix &is, const ComplexMatrix &ds)

Create a complex fixed point matrix with the number of bits in the integer part of each element represented by is and the number in the decimal part by ds. The fixed point elements themselves will be initialized to zero.

FixedComplexMatrix::FixedComplexMatrix (unsigned int is, unsigned int ds, const FixedComplexMatrix& a)
FixedComplexMatrix::FixedComplexMatrix (Complex is, Complex ds, const FixedComplexMatrix& a)

FixedComplexMatrix::FixedComplexMatrix (const MArray2<int> &is, const MArray2<int> &ds, const FixedComplexMatrix& a)
FixedComplexMatrix::FixedComplexMatrix (const Matrix &is, const Matrix &ds, const FixedComplexMatrix& a)

Create a complex fixed point matrix with the number of bits in the integer part of the real and imaginary part of each element represented by is and the number in the decimal part by ds. The fixed point elements themselves will be initialized to the complex fixed point matrix a

`FixedComplexMatrix::FixedComplexMatrix (const ComplexMatrix &is, const ComplexMatrix &ds, const FixedComplexMatrix &x)`
`FixedComplexMatrix::FixedComplexMatrix (const ComplexMatrix &is, const ComplexMatrix &ds, const FixedMatrix &r, const FixedMatrix &i)`

Create a complex fixed point matrix with the number of bits in the integer part of each element represented by `is` and the number in the decimal part by `ds`. The fixed point elements themselves will be initialized to the complex fixed point matrix `a`, or the real part by `r` and the imaginary by `i`.

`FixedComplexMatrix::FixedComplexMatrix (unsigned int is, unsigned int ds, const FixedMatrix& a)`
`FixedComplexMatrix::FixedComplexMatrix (Complex is, Complex ds, const FixedMatrix& a)`

`FixedComplexMatrix::FixedComplexMatrix (const MArray2<int> &is, const MArray2<int> &ds, const FixedMatrix& a)`
`FixedComplexMatrix::FixedComplexMatrix (const Matrix &is, const Matrix &ds, const FixedMatrix& a)`

Create a complex fixed point matrix with the number of bits in the integer part of the real and imaginary part of each element represented by `is` and the number in the decimal part by `ds`. The fixed point elements themselves will be initialized to the fixed point matrix `a`

`FixedComplexMatrix::FixedComplexMatrix (const ComplexMatrix &is, const ComplexMatrix &ds, const FixedMatrix &x)`

Create a complex fixed point matrix with the number of bits in the integer part of each element represented by `is` and the number in the decimal part by `ds`. The fixed point elements themselves will be initialized to the fixed point matrix `a`.

`FixedComplexMatrix::FixedComplexMatrix (unsigned int is, unsigned int ds, const ComplexMatrix& a)`
`FixedComplexMatrix::FixedComplexMatrix (Complex is, Complex ds, const ComplexMatrix& a)`
`FixedComplexMatrix::FixedComplexMatrix (const MArray2<int> &is, const MArray2<int> &ds, const ComplexMatrix& a)`
`FixedComplexMatrix::FixedComplexMatrix (const ComplexMatrix &is, const ComplexMatrix &ds, const ComplexMatrix& a)`

Create a complex fixed point matrix with the number of bits in the integer part of the real and imaginary part of each element represented by `is` and the number in the decimal part by `ds`. The fixed point elements themselves will be initialized to the complex matrix `a`

`FixedComplexMatrix::FixedComplexMatrix (const ComplexMatrix &is, const ComplexMatrix &ds, const ComplexMatrix &x)`
`FixedComplexMatrix::FixedComplexMatrix (const ComplexMatrix &is, const ComplexMatrix &ds, const Matrix &r, const Matrix &i)`

Create a complex fixed point matrix with the number of bits in the integer part of each element represented by `is` and the number in the decimal part by `ds`.

The fixed point elements themselves will be initialized to the complex matrix a, or the real part by r and the imaginary by i.

FixedComplexMatrix::FixedComplexMatrix (unsigned int is, unsigned int ds, const Matrix& a)
FixedComplexMatrix::FixedComplexMatrix (Complex is, Complex ds, const Matrix& a)
FixedComplexMatrix::FixedComplexMatrix (const MArray2<int> &is, const MArray2<int> &ds, const Matrix& a)
FixedComplexMatrix::FixedComplexMatrix (const ComplexMatrix &is, const ComplexMatrix &ds, const Matrix& a)

Create a complex fixed point matrix with the number of bits in the integer part of the real and imaginary part of each element represented by is and the number in the decimal part by ds. The fixed point elements themselves will be initialized to the matrix a

FixedComplexMatrix::FixedComplexMatrix (const ComplexMatrix &is, const ComplexMatrix &ds, const Matrix &x)

Create a complex fixed point matrix with the number of bits in the integer part of each element represented by is and the number in the decimal part by ds. The fixed point elements themselves will be initialized to the matrix a.

FixedComplexMatrix::FixedComplexMatrix (unsigned int is, unsigned int ds, const ComplexMatrix& a, const ComplexMatrix& b)
FixedComplexMatrix::FixedComplexMatrix (Complex is, Complex ds, const ComplexMatrix& a, const ComplexMatrix& b)
FixedComplexMatrix::FixedComplexMatrix (const MArray2<int> &is, const MArray2<int> &ds, const ComplexMatrix& a, const ComplexMatrix& b)
FixedComplexMatrix::FixedComplexMatrix (const Matrix &is, const Matrix &ds, const ComplexMatrix& a, const ComplexMatrix& b)
FixedComplexMatrix::FixedComplexMatrix (const ComplexMatrix &is, const ComplexMatrix &ds, const ComplexMatrix& a, const ComplexMatrix& b)

Create a fixed point matrix with the number of bits in the integer part of each element represented by is and the number in the decimal part by ds.The fixed point elements themselves will have the integer parts loaded by the complex matrix a and the decimal part by the complex matrix b. It should be noted that a and b are both considered as unsigned complex integers and are the strict representation of the bits of the fixed point value.

ComplexMatrix FixedComplexMatrix::fixedpoint ()

Method to create a Matrix from a fixed point matrix x

ComplexMatrix fixedpoint (const FixedComplexMatrix &x)

Create a Matrix from a fixed point matrix x

ComplexMatrix FixedComplexMatrix::sign ()

Return -1 for negative numbers, 1 for positive and 0 if the fixed point element is zero, for every element of the current fixed point object.

`ComplexMatrix sign (const FixedComplexMatrix &x)`
: Return `-1` for negative numbers, `1` for positive and `0` if zero, for every element of the fixed point object `x`.

`ComplexMatrix FixedComplexMatrix::getintsize ()`
: Return the number of bit `is` used to represent the integer part of each element of the current fixed point object.

`ComplexMatrix getintsize (const FixedComplexMatrix &x)`
: Return the number of bit `is` used to represent the integer part of each element of the fixed point object `x`.

`ComplexMatrix FixedComplexMatrix::getdecsize ()`
: Return the number of bit `ds` used to represent the decimal part of each element of the current fixed point object.

`ComplexMatrix getdecsize (const FixedComplexMatrix &x)`
: Return the number of bit `ds` used to represent the decimal part of each element of the fixed point object `x`.

`ComplexMatrix FixedComplexMatrix::getnumber ()`
: Return the integer representation of the fixed point value of the each eleement of the current fixed point object.

`ComplexMatrix getnumber (const FixedComplexMatrix &x)`
: Return the integer representation of the fixed point value of the each eleement of the fixed point object `x`.

`FixedComplexMatrix FixedComplexMatrix::chintsize (const ComplexMatrix &n)`
`FixedComplexMatrix FixedComplexMatrix::chintsize (const double n)`
: Return a fixed point object equivalent to the current fixed point object but with the number of bits representing the integer part `is` of every element changed to `n`. If the result of this operation causes any element of `is + ds` to be greater than `sizeof(int)*8 - 2`, the error handler is called.

`FixedComplexMatrix FixedComplexMatrix::chdecsize (const double n)`
`FixedComplexMatrix FixedComplexMatrix::chdecsize (const ComplexMatrix &n)`
: Return a fixed point object equivalent to the current fixed point object but with the number of bits representing the decimal part `ds` of every element changed to `n`. If the result of this operation causes any element of `is + ds` to be greater than `sizeof(int)*8 - 2`, the error handler is called.

`FixedComplexMatrix FixedComplexMatrix::incintsize (const double n)`
`FixedComplexMatrix FixedComplexMatrix::incintsize (const ComplexMatrix &n)`
: Return a fixed point object equivalent to the current fixed point number but with the number of bits representing the integer part `is` increased by `n`. If `n` is negative, then `is` is decreased. If the result of this operation causes `is + ds` to be greater than `sizeof(int)*8 - 2`, the error handler is called.

`FixedComplexMatrix FixedComplexMatrix::incintsize ()`
: Return a fixed point object equivalent to the current fixed point number but with the number of bits representing the integer part `is` increased by 1. If the

result of this operation causes `is + ds` to be greater than `sizeof(int)*8 - 2`, the error handler is called.

`FixedComplexMatrix FixedComplexMatrix::incdecsize (const double n)`
`FixedComplexMatrix FixedComplexMatrix::incdecsize (const ComplexMatrix &n)`

Return a fixed point object equivalent to the current fixed point number but with the number of bits representing the decimal part `ds` increased by `n`. If `n` is negative, then `ds` is decreased. If the result of this operation causes `is + ds` to be greater than `sizeof(int)*8 - 2`, the error handler is called.

`FixedComplexMatrix FixedComplex::incdecsize ()`

Return a fixed point object equivalent to the current fixed point number but with the number of bits representing the decimal part `ds` increased by 1. If the result of this operation causes `is + ds` to be greater than `sizeof(int)*8 - 2`, the error handler is called.

2.3.5 `FixedComplexRowVector` class

`FixedComplexRowVector::FixedComplexRowVector (const MArray<int> &is, const MArray<int> &ds)`
`FixedComplexRowVector::FixedComplexRowVector (const RowVector &is, const RowVector &ds)`

Create a complex fixed point row vector with the number of bits in the integer part of the real and imaginary part of each element represented by `is` and the number in the decimal part by `ds`. The fixed point elements themselves will be initialized to zero.

`FixedComplexRowVector::FixedComplexRowVector (const ComplexRowVector &is, const ComplexRowVector &ds)`

Create a complex fixed point row vector with the number of bits in the integer part of each element represented by `is` and the number in the decimal part by `ds`. The fixed point elements themselves will be initialized to zero.

`FixedComplexRowVector::FixedComplexRowVector (unsigned int is, unsigned int ds, const FixedComplexRowVector& a)`
`FixedComplexRowVector::FixedComplexRowVector (Complex is, Complex ds, const FixedComplexRowVector& a)`
`FixedComplexRowVector::FixedComplexRowVector (const MArray<int> &is, const MArray<int> &ds, const FixedComplexRowVector& a)`
`FixedComplexRowVector::FixedComplexRowVector (const RowVector &is, const RowVector &ds, const FixedComplexRowVector& a)`

Create a complex fixed point row vector with the number of bits in the integer part of the real and imaginary part of each element represented by `is` and the number in the decimal part by `ds`. The fixed point elements themselves will be initialized to the complex fixed point row vector `a`

FixedComplexRowVector::FixedComplexRowVector (const ComplexRowVector &is, const ComplexRowVector &ds, const FixedComplexRowVector &x)
FixedComplexRowVector::FixedComplexRowVector (const ComplexRowVector &is, const ComplexRowVector &ds, const FixedRowVector &r, const FixedRowVector &i)

Create a complex fixed point row vector with the number of bits in the integer part of each element represented by `is` and the number in the decimal part by `ds`. The fixed point elements themselves will be initialized to the complex fixed point row vector `a`, or the real part by `r` and the imaginary by `i`.

FixedComplexRowVector::FixedComplexRowVector (unsigned int is, unsigned int ds, const FixedRowVector& a)
FixedComplexRowVector::FixedComplexRowVector (Complex is, Complex ds, const FixedRowVector& a)
FixedComplexRowVector::FixedComplexRowVector (const MArray<int> &is, const MArray<int> &ds, const FixedRowVector& a)
FixedComplexRowVector::FixedComplexRowVector (const RowVector &is, const RowVector &ds, const FixedRowVector& a)

Create a complex fixed point row vector with the number of bits in the integer part of the real and imaginary part of each element represented by `is` and the number in the decimal part by `ds`. The fixed point elements themselves will be initialized to the fixed point row vector `a`

FixedComplexRowVector::FixedComplexRowVector (const ComplexRowVector &is, const ComplexRowVector &ds, const FixedRowVector &x)

Create a complex fixed point row vector with the number of bits in the integer part of each element represented by `is` and the number in the decimal part by `ds`. The fixed point elements themselves will be initialized to the fixed point row vector `a`.

FixedComplexRowVector::FixedComplexRowVector (unsigned int is, unsigned int ds, const ComplexRowVector& a)
FixedComplexRowVector::FixedComplexRowVector (Complex is, Complex ds, const ComplexRowVector& a)
FixedComplexRowVector::FixedComplexRowVector (const MArray<int> &is, const MArray<int> &ds, const ComplexRowVector& a)
FixedComplexRowVector::FixedComplexRowVector (const ComplexRowVector &is, const ComplexRowVector &ds, const ComplexRowVector& a)

Create a complex fixed point row vector with the number of bits in the integer part of the real and imaginary part of each element represented by `is` and the number in the decimal part by `ds`. The fixed point elements themselves will be initialized to the complex row vector `a`

FixedComplexRowVector::FixedComplexRowVector (const ComplexRowVector &is, const ComplexRowVector &ds, const ComplexRowVector &x)
FixedComplexRowVector::FixedComplexRowVector (const ComplexRowVector &is, const ComplexRowVector &ds, const RowVector &r, const RowVector &i)

Create a complex fixed point row vector with the number of bits in the integer part of each element represented by `is` and the number in the decimal part by

ds. The fixed point elements themselves will be initialized to the complex row vector a, or the real part by r and the imaginary by i.

FixedComplexRowVector::FixedComplexRowVector (unsigned int is, unsigned int ds, const RowVector& a)
FixedComplexRowVector::FixedComplexRowVector (Complex is, Complex ds, const RowVector& a)
FixedComplexRowVector::FixedComplexRowVector (const MArray<int> &is, const MArray<int> &ds, const RowVector& a)
FixedComplexRowVector::FixedComplexRowVector (const ComplexRowVector &is, const ComplexRowVector &ds, const RowVector& a)

Create a complex fixed point row vector with the number of bits in the integer part of the real and imaginary part of each element represented by is and the number in the decimal part by ds. The fixed point elements themselves will be initialized to the row vector a

FixedComplexRowVector::FixedComplexRowVector (const ComplexRowVector &is, const ComplexRowVector &ds, const RowVector &x)

Create a complex fixed point row vector with the number of bits in the integer part of each element represented by is and the number in the decimal part by ds. The fixed point elements themselves will be initialized to the row vector a.

FixedComplexRowVector::FixedComplexRowVector (unsigned int is, unsigned int ds, const ComplexRowVector& a, const ComplexRowVector& b)
FixedComplexRowVector::FixedComplexRowVector (Complex is, Complex ds, const ComplexRowVector& a, const ComplexRowVector& b)
FixedComplexRowVector::FixedComplexRowVector (const MArray<int> &is, const MArray<int> &ds, const ComplexRowVector& a, const ComplexRowVector& b)
FixedComplexRowVector::FixedComplexRowVector (const RowVector &is, const RowVector &ds, const ComplexRowVector& a, const ComplexRowVector& b)
FixedComplexRowVector::FixedComplexRowVector (const ComplexRowVector &is, const ComplexRowVector &ds, const ComplexRowVector& a, const ComplexRowVector& b)

Create a fixed point row vector with the number of bits in the integer part of each element represented by is and the number in the decimal part by ds. The fixed point elements themselves will have the integer parts loaded by the complex row vector a and the decimal part by the complex row vector b. It should be noted that a and b are both considered as unsigned complex integers and are the strict representation of the bits of the fixed point value.

ComplexRowVector FixedComplexRowVector::fixedpoint ()

Method to create a RowVector from a fixed point row vector x

ComplexRowVector fixedpoint (const FixedComplexRowVector &x)

Create a RowVector from a fixed point row vector x

ComplexRowVector FixedComplexRowVector::sign ()

Return -1 for negative numbers, 1 for positive and 0 if the fixed point element is zero, for every element of the current fixed point object.

`ComplexRowVector sign (const FixedComplexRowVector &x)`
Return -1 for negative numbers, 1 for positive and 0 if zero, for every element of the fixed point object x.

`ComplexRowVector FixedComplexRowVector::getintsize ()`
Return the number of bit is used to represent the integer part of each element of the current fixed point object.

`ComplexRowVector getintsize (const FixedComplexRowVector &x)`
Return the number of bit is used to represent the integer part of each element of the fixed point object x.

`ComplexRowVector FixedComplexRowVector::getdecsize ()`
Return the number of bit ds used to represent the decimal part of each element of the current fixed point object.

`ComplexRowVector getdecsize (const FixedComplexRowVector &x)`
Return the number of bit ds used to represent the decimal part of each element of the fixed point object x.

`ComplexRowVector getdecsize (const FixedComplexRowVector &x)`
Return the number of bit ds used to represent the decimal part of each element of the fixed point object x.

`ComplexRowVector FixedComplexRowVector::getnumber ()`
Return the integer representation of the fixed point value of the each eleement of the current fixed point object.

`FixedComplexRowVector FixedComplexRowVector::chintsize (const ComplexRowVector &n)`
`FixedComplexRowVector FixedComplexRowVector::chintsize (const double n)`
Return a fixed point object equivalent to the current fixed point object but with the number of bits representing the integer part is of every element changed to n. If the result of this operation causes any element of is + ds to be greater than sizeof(int)*8 - 2, the error handler is called.

`FixedComplexRowVector FixedComplexRowVector::chdecsize (const double n)`
`FixedComplexRowVector FixedComplexRowVector::chdecsize (const ComplexRowVector &n)`
Return a fixed point object equivalent to the current fixed point object but with the number of bits representing the decimal part ds of every element changed to n. If the result of this operation causes any element of is + ds to be greater than sizeof(int)*8 - 2, the error handler is called.

`FixedComplexRowVector FixedComplexRowVector::incintsize (const Complex n)`
`FixedComplexRowVector FixedComplexRowVector::incintsize (const ComplexRowVector &n)`
Return a fixed point object equivalent to the current fixed point number but with the number of bits representing the integer part is increased by n. If n is negative, then is is decreased. If the result of this operation causes is + ds to be greater than sizeof(int)*8 - 2, the error handler is called.

`FixedComplexRowVector FixedComplexRowVector::incintsize ()`

Return a fixed point object equivalent to the current fixed point number but with the number of bits representing the integer part `is` increased by 1. If the result of this operation causes `is + ds` to be greater than `sizeof(int)*8 - 2`, the error handler is called.

`FixedComplexRowVector FixedComplexRowVector::incdecsize (const Complex n)`
`FixedComplexRowVector FixedComplexRowVector::incdecsize (const ComplexRowVector &n)`

Return a fixed point object equivalent to the current fixed point number but with the number of bits representing the decimal part `ds` increased by `n`. If `n` is negative, then `ds` is decreased. If the result of this operation causes `is + ds` to be greater than `sizeof(int)*8 - 2`, the error handler is called.

`FixedComplexRowVector FixedComplexRowVector::incdecsize ()`

Return a fixed point object equivalent to the current fixed point number but with the number of bits representing the decimal part `ds` increased by 1. If the result of this operation causes `is + ds` to be greater than `sizeof(int)*8 - 2`, the error handler is called.

2.3.6 `FixedComplexColumnVector` class

`FixedComplexColumnVector::FixedComplexColumnVector (const MArray<int> &is, const MArray<int> &ds)`
`FixedComplexColumnVector::FixedComplexColumnVector (const ColumnVector &is, const ColumnVector &ds)`

Create a complex fixed point column vector with the number of bits in the integer part of the real and imaginary part of each element represented by `is` and the number in the decimal part by `ds`. The fixed point elements themselves will be initialized to zero.

`FixedComplexColumnVector::FixedComplexColumnVector (const ComplexColumnVector &is, const ComplexColumnVector &ds)`

Create a complex fixed point column vector with the number of bits in the integer part of each element represented by `is` and the number in the decimal part by `ds`. The fixed point elements themselves will be initialized to zero.

`FixedComplexColumnVector::FixedComplexColumnVector (unsigned int is, unsigned int ds, const FixedComplexColumnVector& a)`
`FixedComplexColumnVector::FixedComplexColumnVector (Complex is, Complex ds, const FixedComplexColumnVector& a)`
`FixedComplexColumnVector::FixedComplexColumnVector (const MArray<int> &is, const MArray<int> &ds, const FixedComplexColumnVector& a)`
`FixedComplexColumnVector::FixedComplexColumnVector (const ColumnVector &is, const ColumnVector &ds, const FixedComplexColumnVector& a)`

Create a complex fixed point column vector with the number of bits in the integer part of the real and imaginary part of each element represented by `is` and the number in the decimal part by `ds`. The fixed point elements themselves will be initialized to the complex fixed point column vector `a`

FixedComplexColumnVector::FixedComplexColumnVector (const ComplexColumnVector &is, const ComplexColumnVector &ds, const FixedComplexColumnVector &x)
FixedComplexColumnVector::FixedComplexColumnVector (const ComplexColumnVector &is, const ComplexColumnVector &ds, const FixedColumnVector &r, const FixedColumnVector &i)

Create a complex fixed point column vector with the number of bits in the integer part of each element represented by is and the number in the decimal part by ds. The fixed point elements themselves will be initialized to the complex fixed point column vector a, or the real part by r and the imaginary by i.

FixedComplexColumnVector::FixedComplexColumnVector (unsigned int is, unsigned int ds, const FixedColumnVector& a)
FixedComplexColumnVector::FixedComplexColumnVector (Complex is, Complex ds, const FixedColumnVector& a)
FixedComplexColumnVector::FixedComplexColumnVector (const MArray<int> &is, const MArray<int> &ds, const FixedColumnVector& a)
FixedComplexColumnVector::FixedComplexColumnVector (const ColumnVector &is, const ColumnVector &ds, const FixedColumnVector& a)

Create a complex fixed point column vector with the number of bits in the integer part of the real and imaginary part of each element represented by is and the number in the decimal part by ds. The fixed point elements themselves will be initialized to the fixed point column vector a

FixedComplexColumnVector::FixedComplexColumnVector (const ComplexColumnVector &is, const ComplexColumnVector &ds, const FixedColumnVector &x)

Create a complex fixed point column vector with the number of bits in the integer part of each element represented by is and the number in the decimal part by ds. The fixed point elements themselves will be initialized to the fixed point column vector a.

FixedComplexColumnVector::FixedComplexColumnVector (unsigned int is, unsigned int ds, const ComplexColumnVector& a)
FixedComplexColumnVector::FixedComplexColumnVector (Complex is, Complex ds, const ComplexColumnVector& a)
FixedComplexColumnVector::FixedComplexColumnVector (const MArray<int> &is, const MArray<int> &ds, const ComplexColumnVector& a)
FixedComplexColumnVector::FixedComplexColumnVector (const ComplexColumnVector &is, const ComplexColumnVector &ds, const ComplexColumnVector& a)

Create a complex fixed point column vector with the number of bits in the integer part of the real and imaginary part of each element represented by is and the number in the decimal part by ds. The fixed point elements themselves will be initialized to the complex column vector a

`FixedComplexColumnVector::FixedComplexColumnVector (const ComplexColumnVector &is, const ComplexColumnVector &ds, const ComplexColumnVector &x)`
`FixedComplexColumnVector::FixedComplexColumnVector (const ComplexColumnVector &is, const ComplexColumnVector &ds, const ColumnVector &r, const ColumnVector &i)`

> Create a complex fixed point column vector with the number of bits in the integer part of each element represented by `is` and the number in the decimal part by `ds`. The fixed point elements themselves will be initialized to the complex column vector `a`, or the real part by `r` and the imaginary by `i`.

`FixedComplexColumnVector::FixedComplexColumnVector (unsigned int is, unsigned int ds, const ColumnVector& a)`
`FixedComplexColumnVector::FixedComplexColumnVector (Complex is, Complex ds, const ColumnVector& a)`
`FixedComplexColumnVector::FixedComplexColumnVector (const MArray<int> &is, const MArray<int> &ds, const ColumnVector& a)`
`FixedComplexColumnVector::FixedComplexColumnVector (const ComplexColumnVector &is, const ComplexColumnVector &ds, const ColumnVector& a)`

> Create a complex fixed point column vector with the number of bits in the integer part of the real and imaginary part of each element represented by `is` and the number in the decimal part by `ds`. The fixed point elements themselves will be initialized to the column vector `a`

`FixedComplexColumnVector::FixedComplexColumnVector (const ComplexColumnVector &is, const ComplexColumnVector &ds, const ColumnVector &x)`

> Create a complex fixed point column vector with the number of bits in the integer part of each element represented by `is` and the number in the decimal part by `ds`. The fixed point elements themselves will be initialized to the column vector `a`.

`FixedComplexColumnVector::FixedComplexColumnVector (unsigned int is, unsigned int ds, const ComplexColumnVector& a, const ComplexColumnVector& b)`
`FixedComplexColumnVector::FixedComplexColumnVector (Complex is, Complex ds, const ComplexColumnVector& a, const ComplexColumnVector& b)`
`FixedComplexColumnVector::FixedComplexColumnVector (const MArray<int> &is, const MArray<int> &ds, const ComplexColumnVector& a, const ComplexColumnVector& b)`
`FixedComplexColumnVector::FixedComplexColumnVector (const ColumnVector &is, const ColumnVector &ds, const ComplexColumnVector& a, const ComplexColumnVector& b)`
`FixedComplexColumnVector::FixedComplexColumnVector (const ComplexColumnVector &is, const ComplexColumnVector &ds, const ComplexColumnVector& a, const ComplexColumnVector& b)`

> Create a fixed point column vector with the number of bits in the integer part of each element represented by `is` and the number in the decimal part by `ds`.

The fixed point elements themselves will have the integer parts loaded by the complex column vector `a` and the decimal part by the compelx column vector `b`. It should be noted that `a` and `b` are both considered as unsigned compelx integers and are the strict representation of the bits of the fixed point value.

`ComplexColumnVector FixedComplexColumnVector::fixedpoint ()`
: Method to create a `ColumnVector` from a fixed point column vector `x`

`ComplexColumnVector fixedpoint (const FixedComplexColumnVector &x)`
: Create a `ColumnVector` from a fixed point column vector `x`

`ComplexColumnVector FixedComplexColumnVector::sign ()`
: Return `-1` for negative numbers, `1` for positive and `0` if the fixed point element is zero, for every element of the current fixed point object.

`ComplexColumnVector sign (const FixedComplexColumnVector &x)`
: Return `-1` for negative numbers, `1` for positive and `0` if zero, for every element of the fixed point object `x`.

`ComplexColumnVector FixedComplexColumnVector::getintsize ()`
: Return the number of bit `is` used to represent the integer part of each element of the current fixed point object.

`ComplexColumnVector getintsize (const FixedComplexColumnVector &x)`
: Return the number of bit `is` used to represent the integer part of each element of the fixed point object `x`.

`ComplexColumnVector FixedComplexColumnVector::getdecsize ()`
: Return the number of bit `ds` used to represent the decimal part of each element of the current fixed point object.

`ComplexColumnVector getdecsize (const FixedComplexColumnVector &x)`
: Return the number of bit `ds` used to represent the decimal part of each element of the fixed point object `x`.

`ComplexColumnVector FixedComplexColumnVector::getnumber ()`
: Return the integer representation of the fixed point value of the each eleement of the current fixed point object.

`ComplexColumnVector getnumber (const FixedComplexColumnVector &x)`
: Return the integer representation of the fixed point value of the each eleement of the fixed point object `x`.

`FixedComplexColumnVector FixedComplexColumnVector::chintsize (const ComplexColumnVector &n)`
`FixedComplexColumnVector FixedComplexColumnVector::chintsize (const double n)`
: Return a fixed point object equivalent to the current fixed point object but with the number of bits representing the integer part `is` of every element changed to `n`. If the result of this operation causes any element of `is + ds` to be greater than `sizeof(int)*8 - 2`, the error handler is called.

`FixedComplexColumnVector FixedComplexColumnVector::chdecsize (const double n)`
`FixedComplexColumnVector FixedComplexColumnVector::chdecsize (const ComplexColumnVector &n)`

Return a fixed point object equivalent to the current fixed point object but with the number of bits representing the decimal part `ds` of every element changed to `n`. If the result of this operation causes any element of `is + ds` to be greater than `sizeof(int)*8 - 2`, the error handler is called.

`FixedComplexColumnVector FixedComplexColumnVector::incintsize (const double n)`
`FixedComplexColumnVector FixedComplexColumnVector::incintsize (const ComplexColumnVector &n)`

Return a fixed point object equivalent to the current fixed point number but with the number of bits representing the integer part `is` increased by `n`. If `n` is negative, then `is` is decreased. If the result of this operation causes `is + ds` to be greater than `sizeof(int)*8 - 2`, the error handler is called.

`FixedComplexColumnVector FixedComplexColumnVector::incintsize ()`

Return a fixed point object equivalent to the current fixed point number but with the number of bits representing the integer part `is` increased by 1. If the result of this operation causes `is + ds` to be greater than `sizeof(int)*8 - 2`, the error handler is called.

`FixedComplexColumnVector FixedComplexColumnVector::incdecsize (const double n)`
`FixedComplexColumnVector FixedComplexColumnVector::incdecsize (const ComplexColumnVector &n)`

Return a fixed point object equivalent to the current fixed point number but with the number of bits representing the decimal part `ds` increased by `n`. If `n` is negative, then `ds` is decreased. If the result of this operation causes `is + ds` to be greater than `sizeof(int)*8 - 2`, the error handler is called.

`FixedComplexColumnVector FixedComplexColumnVector::incdecsize ()`

Return a fixed point object equivalent to the current fixed point number but with the number of bits representing the decimal part `ds` increased by 1. If the result of this operation causes `is + ds` to be greater than `sizeof(int)*8 - 2`, the error handler is called.

2.4 The Upper Level Octave Classes

There are 4 upper level classes that define the interface of the fixed point type to the octave interpreter. These mirror similar definitions for floating values in octave and are

Fixed Point	Floating Point
octave_fixed	octave_scalar
octave_fixed_complex	octave_complex
octave_fixed_matrix	octave_matrix
octave_fixed_complex_matrix	octave_complex_matrix

These fixed point classes use the same base classes as the corresponding floating classes, and so have similar capabilities. However, one notable addition are the methods to obtain the base fixed point objects from these classes, and also correspond to similar floating point methods. These are

Fixed Point	Floating Point
`fixed_value`	`scalar_value`
`fixed_complex_value`	`complex_value`
`fixed_matrix_value`	`matrix_value`
`fixed_complex_matrix_value`	`complex_matrix_value`

and can be used in all fixed point classes, subject to a possible reduction operations (eg. casts a `FixedComplexMatrix` as a `FixedPoint`). In addition the methods `scalar_value`, `complex_value`, `matrix_value` and `complex_matrix_value` can all be used.

The user should examine the files ‘`ov-fixed-cx-mat.h`’, ‘`ov-fixed-complex.h`’, ‘`ov-fixed-mat.h`’ and ‘`ov-fixed.h`’ and the base classes in the files ‘`ov-base-fixed.h`’, ‘`ov-base-fixed-mat.h`’, and file ‘`ov-base.h`’ within octave to see the methods that are available for the upper level classes.

2.5 Writing Oct-files with the Fixed Point Type

It is not the purpose of this section to discuss how to write an oct-file, or discuss what they are, but rather the specifics of using oct-files with the fixed point type. An oct-file is a means of writing an octave function in a compilable language like C++, rather than as a script file. This results in a significant acceleration in the code. The reader is referred to the tutorial available at `http://octave.sourceforge.net/coda/coda.html`. The examples discussed here assume that the oct-file is written entirely in C++.

2.5.1 Using C++ Templates in Oct-files

When using the fixed point toolbox, you will almost certainly want to compare an implementation in fixed-point against the corresponding implementation in floating point. This allows the degradation due to the conversion to a fixed-point algorithm to be easily observed. The concept of C++ templates allows easy implementation of both fixed and floating point versions of the same code. For example consider

```
template <class A, class B>
A myfunc(const A &a, const B &b) {
  return (a + b);
}

// Floating point instantiations
template double myfunc (const double&, const double&);
template Complex myfunc (const Complex&, const double&);
template Matrix myfunc (const Matrix&, const double&);
template ComplexMatrix myfunc (const ComplexMatrix&, const double&);

// Fixed point instantiations
```

```
template FixedPoint myfunc (const FixedPoint&, const FixedPoint&);
template FixedPointComplex myfunc (const FixedPointComplex&,
   const FixedPoint&);
template FixedMatrix myfunc (const FixedMatrix&, const FixedPoint&);
template FixedComplexMatrix myfunc (const FixedComplexMatrix&,
    const FixedPoint&);
```

Eight versions of the function `myfunc` are created, that allow its use with all floating and fixed types.

2.5.2 Specific Problems of Oct-files using Fixed Point

The fact that the fixed point type is loadable, means that the symbols of this type are not available till the first use of a fixed point variable. This means that the function *fixed* must be called at least once prior to accessing fixed point values in an oct-file. If a user function is called, that uses a fixed point type, before the type is loaded the result will be an error message complaining of unknown symbols. For example

```
octave:1> fixed_inc(a)
error: /home/dbateman/octave/fixed/fixed_inc.oct: undefined symbol:
_ZN24octave_base_fixed_matrixI18FixedComplexMatrixE7subsref
ERKSsRKSt4listI17octave_value_listSaIS5_EE
error: ‘fixed_inc’ undefined near line 1 column 1
```

This should not in itself result in an abnormal exit from Octave.

Another problem when accessing fixed point variables within oct-files, is that the Octave `octave_value` class knows nothing about this type. So you can not directly call a method such as `fixed_value()` on the input arguments, but rather must cast the representation of the `octave_value` as a fixed type and then call the relevant method. An example of extracting a fixed point value from an `octave_value` variable `arg` is then

```
octave_value arg;
...
if (arg.type_id () == octave_fixed::static_type_id ()) {
  FixedPoint f = ((const octave_fixed&) arg.get_rep()).fixed_value();
```

Similarly, the return value from an oct-file is itself either an `octave_value` or an `octave_value_list`. So a special means of creating the return value must be used. For example

```
octave_value_list retval;
Matrix m;
FixedPoint f;
...
retval(0) = octave_value(m);
retval(1) = new octave_fixed (f);
```

2.5.3 Specific points to note when using Oct-files and Cygwin

When using the GNU C++ compiler under Cygwin to create a shared object, such as an oct-file, the symbols must be resolved at compile time. This is as opposed to a Unix platform where the resolution of the symbols can be left till runtime. For this reason the fixed point type, when built under Cygwin is split into an oct-file and a shared library called

'liboctave_fixed.dll'. This is as opposed to the situation under Unix, where only the Oct-file containing all of the fixed point type is needed.

This has the implication that when you build an Oct-file under Cygwin using the fixed point type, that they must be linked to 'liboctave_fixed.dll'. An appropriate means to do this is for example

```
% mkoctfile -loctave_fixed myfile.cc
```

The file 'liboctave_fixed.dll' and 'liboctave_fixed.dll.a' must be located somewhere that the compiler can find them. If these are installed in the same directory as 'liboctave.dll' and 'liboctave.dll.a' respectively, then mkoctfile will find them automatically.

2.5.4 A Simple Example of an Oct-file

An example of a simple oct-file written in C++ using the fixed point type can be seen below. It integrates the ideas discussed previously.

```
#include <octave/config.h>
#include <octave/oct.h>
#include "fixed.h"

template <class A, class B>
A myfunc(const A &a, const B &b) {
  return (a + b);
}

// Floating point instantiations
template double myfunc (const double&, const double&);
template Complex myfunc (const Complex&, const double&);
template Matrix myfunc (const Matrix&, const double&);
template ComplexMatrix myfunc (const ComplexMatrix&, const double&);

// Fixed point instantiations
template FixedPoint myfunc (const FixedPoint&, const FixedPoint&);
template FixedPointComplex myfunc (const FixedPointComplex&,
   const FixedPoint&);
template FixedMatrix myfunc (const FixedMatrix&, const FixedPoint&);
template FixedComplexMatrix myfunc (const FixedComplexMatrix&,
    const FixedPoint&);

DEFUN_DLD (fixed_inc, args, ,
"-*- texinfo -*-\n\
@deftypefn {Loadable Function} {@var{y} =}  fixed_inc (@var{x})\n\
Example code of the use of the fixed point types in an oct-file.\n\
Returns @code{@var{x} + 1}\n\
@end deftypefn")
{
  octave_value retval;
  FixedPoint one(1,0,1,0);  // Fixed Point value of 1
```

```
  if (args.length() != 1)
    print_usage("fixed_inc");
  else
    if (args(0).type_id () == octave_fixed_matrix::static_type_id ()) {
      FixedMatrix f = ((const octave_fixed_matrix&) args(0).get_rep()).
        fixed_matrix_value();
      retval = new octave_fixed_matrix (myfunc(f,one));
    } else if (args(0).type_id () == octave_fixed::static_type_id ()) {
      FixedPoint f = ((const octave_fixed&) args(0).get_rep()).
        fixed_value();
      retval = new octave_fixed (myfunc(f,one));
    } else if (args(0).type_id () ==
              octave_fixed_complex::static_type_id ()) {
      FixedPointComplex f = ((const octave_fixed_complex&)
     args(0).get_rep()).fixed_complex_value();
      retval = new octave_fixed_complex (myfunc(f,one));
    } else if (args(0).type_id () ==
              octave_fixed_complex_matrix::static_type_id ()) {
      FixedComplexMatrix f = ((const octave_fixed_complex_matrix&)
              args(0).get_rep()).fixed_complex_matrix_value();
      retval = new octave_fixed_complex_matrix (myfunc(f,one));
    } else {
      // promote the operation to complex matrix. The narrowing op in
      // octave_value will later change the type if needed. This is not
      // optimal but is convenient....
      ComplexMatrix f = args(0).complex_matrix_value();
      retval = octave_value (myfunc(f,1.));
    }
  return retval;
}
```

3 Fixed Point Type Applied to Real Signal Processing Example

As an example of the use of the fixed point toolbox applied to a real signal processing example, we consider the implementation of a Radix-4 IFFT in an OFDM modulator. Code for this IFFT has been written as a C++ template class, and integrated as an Octave Oct-file. This allowed a single version of the code to be instantiated to perform both the fixed and floating point implementations of the same code. The instantiations of this class are

```
template Fft<double,Complex,ComplexRowVector>;
template Fft<FixedPoint,FixedPointComplex,FixedComplexRowVector>;

template Ifft<double,Complex,ComplexRowVector>;
template Ifft<FixedPoint,FixedPointComplex,FixedComplexRowVector>;
```

The code for this example is available as part of the release of this software package.

A particular problem of a hardware implementation of an IFFT is that each butterfly in the radix-4 IFFT consists of the summation of four terms with a suitable phase. Thus, an additional 2 output bits are potentially needed after each butterfly of the radix-4 IFFT. There are several ways of addressing this issue

1. Increase the number of bits in the fixed point representation by two after each radix-4 butterfly. There are then two ways of treating these added bits:

   A. Accept them and let the size of the representation of the fixed point numbers grows. For large IFFT's this is not acceptable

   B. Cut the least significant bits of representation, either after each butterfly, or after groups of butterflies. This reduces the number of bits in the representation, but still trades off complexity to avoid an overflow condition

2. Keep the fixed point representation used in the IFFT fixed, but reduce the input signal level to avoid overflows. The IFFT can then have internal overflows.

An overflow will cause a bit-error which is not necessarily critical. The last option is therefore attractive in that it allows the minimum complexity in the hardware implementation of the IFFT. However, careful investigation of the overflow effects are needed, which can be performed with the fixed point toolbox.

The figures 1 and 2 below shows the case of a 64QAM OFDM signal similar to that used in the 802.11a standard. In this figure the OFDM modulator has been represented using fixed point, while the rest of the system is assumed to be perfect. Figures 1 and 2 shows the tradeoff between the backoff of the RMS power in the frequency domain signal relative to the fixed point representation for several different fixed point representations.

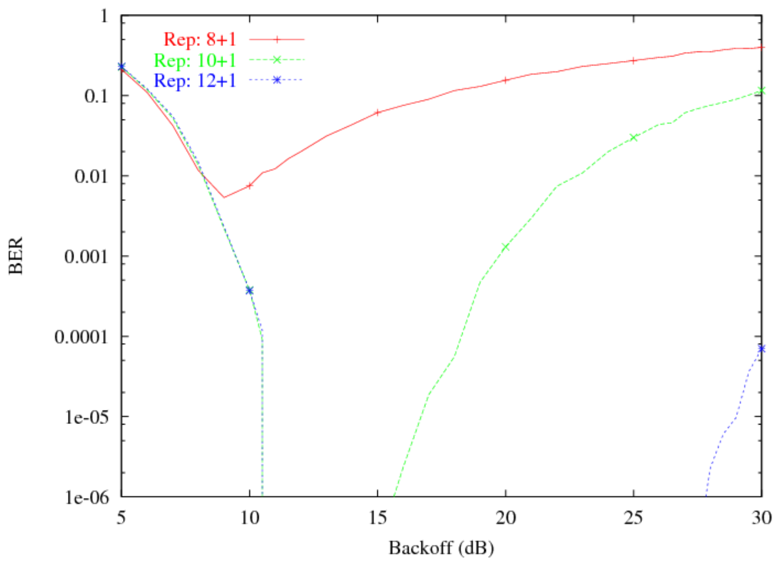

Figure 1: Bit-error rate due to fixed point representation for various backoffs of the RMS power in frequency domain signal. Fixed point representation of n bits plus 1 bit for the sign

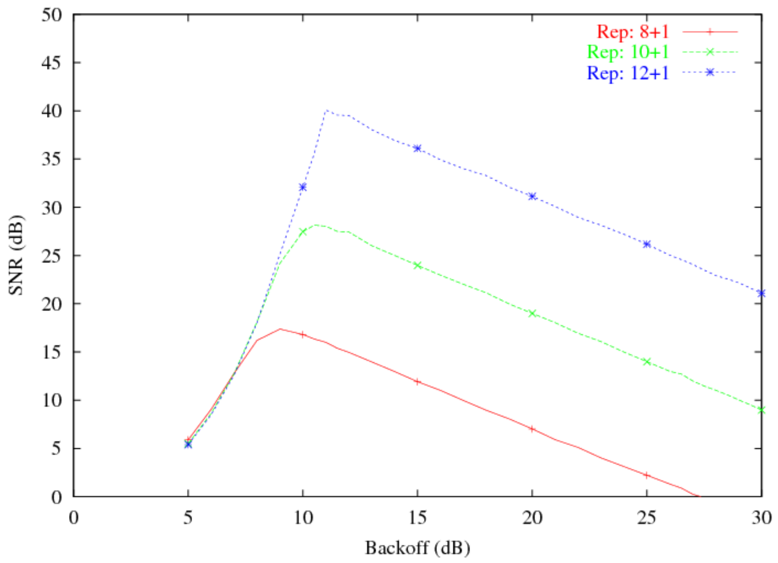

Figure 2: The signal-to-noise ratio as measured by comparing a fixed to a floating point representation for various backoffs of the RMS power in frequency domain signal. Fixed point representation of n bits plus 1 bit for the sign

Two regions are clearly visible in these figures. When the backoff of the RMS power is small, the effects of the overflow in the IFFT dominate, and reduce the performance. When the backoff is large, there are fewer bits in the fixed point representation relative to the average signal power and therefore a slow degradation in the performance. It is clear that somewhere between 11 and 13 bits in the representation of the fixed point numbers in the IFFT is optimal, with a backoff of approximately 13dB.

4 Function Reference

4.1 Functions by Category

4.1.1 Fixed Point Operators

+ - Addition/subtraction in a fixed point algebra.

* / \ Multiplication/division in fixed point (Division for scalars only).

.* ./ .\ Element by element multiplication/division of fixed point arrays.

** ^ Matrix exponentiation of fixed point arrays.

.** .^ Element by element matrix exponentiation of fixed point arrays.

' .' Matrix transpose of fixed point arrays.

== ~= != > >= < <=
Logical operators on fixed point arrays.

4.1.2 Fixed Point Variables

fixed_point_warn_overflow
Query or set the internal variable 'fixed_point_warn_overflow'.

fixed_point_debug
Query or set the internal variable 'fixed_point_debug'.

fixed_point_count_operations
Query or set the internal variable 'fixed_point_count_operations'.

fixed_point_version
A function returning the version number of the fixed point package used.

fixed_point_library_version
A function returning the version number of the fixed point library used.

4.1.3 Fixed Point Utility Functions

concat Concatenate two matrices regardless of their type.

create_lookup_table
Creates a lookup table betwen the vectors X and Y.

display_fixed_operations
Displays out a summary of the number of fixed point operations of each type that have been used.

fdiag Return a diagonal matrix with fixed point vector V on diagonal K.

fixed Used the create a fixed point variable.

fixedpoint Manual and test code for the Octave Fixed Point toolbox.

float Converts a fixed point object to the equivalent floating point object.

freshape Return a fixed matrix with M rows and N columns whose elements are taken from the fixed matrix A.

fsort Return a copy of the fixed point variable X with the elements arranged in increasing order.

isfixed Return 1 if the value of the expression EXPR is a fixed point value.

length *Not implemented*

lookup_table
Using the lookup table created by "create_lookup_table", find the value Y corresponding to X.

reset_fixed_operations
Reset the count of fixed point operations to zero.

size *Not implemented*

all *Not implemented*

any *Not implemented*

4.1.4 Fixed Point Functions

fabs Compute the magnitude of the fixed point value X.

fangle See "farg".

farg Compute the argument of X, defined as THETA = 'atan2 (Y, X)' in radians.

fatan2 Compute atan (Y / X) for corresponding fixed point elements of Y and X.

fceil Return the smallest integer not less than X.

fconj Returns the conjuate of the fixed point value X.

fcosh Compute the hyperbolic cosine of the fixed point value X.

fcos Compute the cosine of the fixed point value X.

fcumprod Cumulative product of elements along dimension DIM.

fcumsum Cumulative sum of elements along dimension DIM.

fexp Compute the exponential of the fixed point value X.

ffloor Return the largest integer not greater than X.

fimag Returns the imaginary part of the fixed point value X.

flog10 Compute the base-10 logarithm of the fixed point value X.

flog Compute the natural logarithm of the fixed point value X.

fprod Product of elements along dimension DIM.

freal Returns the real part of the fixed point value X.

fround Return the rounded value to the nearest integer of X.

fsinh Compute the hyperbolic sine of the fixed point value X.

fsin Compute the sine of the fixed point value X.

fsqrt Compute the square-root of the fixed point value X.

fsum Sum of elements along dimension DIM.

fsumsq Sum of squares of elements along dimension DIM.

ftanh Compute the hyperbolic tan of the fixed point value X.

ftan Compute the tan of the fixed point value X.

4.1.5 Examples

ffft Radix-4 fft in floating and fixed point for vectors of length 4^N, where N is an integer.

fifft Radix-4 ifft in fixed point for vectors of length 4^N, where.

fixed_inc Example code of the use of the fixed point types in an oct-file.

4.2 Functions Alphabetically

4.2.1 concat

x = **concat** (*a*, *b*) Function File

x = **concat** (*a*, *b*, *dim*) Function File

Concatenate two matrices regardless of their type. Due to the implementation of the matrix concatenation in Octave being hard-coded for the types it knowns, user types can not use the matrix concatenation operator. Thus for the *Galois* and *Fixed Point* types, the in-built matrix concatenation functions will return a matrix value as their solution

This function allows these types to be concatenated. If called with a user type that is not known by this function, the in-built concatenate function is used

If *dim* is 1, then the matrices are concatenated, else if *dim* is 2, they are stacked

4.2.2 create_lookup_table

table = **create_lookup_table** (*x*, *y*) Function File

Creates a lookup table betwen the vectors *x* and *y*. If *x* is not in increasing order, the vectors are sorted before being stored

4.2.3 display_fixed_operations

display_fixed_operations ( ) Loadable Function

Displays out a summary of the number of fixed point operations of each type that have been used. This can be used to give a estimate of the complexity of an algorithm.

See also: fixed_point_count_operations, reset_fixed_operations

4.2.4 fabs

y = **fabs** (*x*) Loadable Function

Compute the magnitude of the fixed point value *x*.

4.2.5 fangle

y = **fangle** (*x*) Loadable Function

See *farg*.

4.2.6 farg

y = **farg** (*x*) Loadable Function

Compute the argument of *x*, defined as $\theta = atan2(y, x)$ in radians. For example

```
farg (fixed (3,5,3+4i))
     ⇒  0.90625
```

4.2.7 fatan2

fatan2 (*y*, *x*) Loadable Function

Compute atan (Y / X) for corresponding fixed point elements of Y and X. The result is in range -pi to pi.

4.2.8 fceil

y = **fceil** (*x*) Loadable Function

Return the smallest integer not less than *x*.

See also: fround, ffloor

4.2.9 fconj

y = **fconj** (*x*) Loadable Function

Returns the conjuate of the fixed point value *x*.

4.2.10 fcos

y = **fcos** (*x*) Loadable Function

Compute the cosine of the fixed point value *x*.

See also: fcosh, fsin, fsinh, ftan, ftanh

4.2.11 fcosh

y = **fcosh** (*x*) Loadable Function

Compute the hyperbolic cosine of the fixed point value *x*.

See also: fcos, fsin, fsinh, ftan, ftanh

4.2.12 fcumprod

y = **fcumprod** (*x*, *dim*) Loadable Function

Cumulative product of elements along dimension *dim*. If *dim* is omitted, it defaults to 1 (column-wise cumulative products).

See also: fcumsum

4.2.13 fcumsum

y = **fcumsum** (*x*, *dim*) Loadable Function

Cumulative sum of elements along dimension *dim*. If *dim* is omitted, it defaults to 1 (column-wise cumulative sums).

See also: fcumprod

4.2.14 fdiag

fdiag (*v*, *k*) Loadable Function

Return a diagonal matrix with fixed point vector *v* on diagonal *k*. The second argument is optional. If it is positive, the vector is placed on the *k*-th super-diagonal. If it is negative, it is placed on the -*k*-th sub-diagonal. The default value of *k* is 0, and the vector is placed on the main diagonal. For example,

```
fdiag (fixed(3,2,[1, 2, 3]), 1)
ans =

  0.00  1.00  0.00  0.00
  0.00  0.00  2.00  0.00
  0.00  0.00  0.00  3.00
  0.00  0.00  0.00  0.00
```

Note that if all of the elements of the original vector have the same fixed point representation, then the zero elements in the matrix are created with the same representation. Otherwise the zero elements are created with the equivalent of the fixed point value `fixed(0,0,0)`.n

See also: diag

4.2.15 fexp

y = **fexp** (*x*) Loadable Function

Compute the exponential of the fixed point value *x*.

See also: log, log10, pow

4.2.16 ffft

y = **ffft** (*x*) Loadable Function

Radix-4 fft in floating and fixed point for vectors of length 4^*n*, where *n* is an integer. The variable *x* can be a either a row of column vector, in which case a single fft is carried out over the vector of length 4^*n*. If *x* is a matrix, the fft is carried on each column of *x* and the matrix must contain 4^*n* rows.

The radix-4 fft is implemented in a manner that attempts to approximate how it will be implemented in hardware, rather than use a generic butterfly. The radix-4 algorithm is faster and more precise than the equivalent radix-2 algorithm, and thus is preferred for hardware implementation. See also: fifft

4.2.17 ffloor

y = **ffloor** (*x*) Loadable Function

Return the largest integer not greater than *x*.

See also: fround, fceil

4.2.18 fifft

y = **fifft** (*x*) Loadable Function

Radix-4 ifft in fixed point for vectors of length 4^*n*, where. *n* is an integer. The variable *x* can be a either a row of column vector, in which case a single ifft is carried out over the vector of length 4^*n*. If *x* is a matrix, the ifft is carried on each column of *x* and the matrix must contain 4^*n* rows.

The radix-4 ifft is implemented in a manner that attempts to approximate how it will be implemented in hardware, rather than use a generic butterfly. The radix-4 algorithm is faster and more precise than the equivalent radix-2 algorithm, and thus is preferred for hardware implementation. See also: ffft

4.2.19 fimag

y = **fimag** (*x*) Loadable Function

Returns the imaginary part of the fixed point value *x*.

4.2.20 fixed

y = **fixed** (*f*) Loadable Function
y = **fixed** (*is*, *ds*) Loadable Function
y = **fixed** (*is*, *ds*, *f*) Loadable Function

Used the create a fixed point variable. Called with a single argument, if *f* is itself a fixed point value, then *fixed* is equivalent to *y* = *f*. Otherwise the integer part of *f* is used to create a fixed point variable with the minimum number of bits needed to represent it. *f* can be either real of complex.

Called with two or more arguments *is* represents the number of bits used to represent the integer part of the fixed point numbers, and *ds* the number used to represent the decimal part. These variables must be either positive integer scalars or matrices. If they are matrices they must be of the same dimension, and each fixed point number in the created matrix will have the representation given by the corresponding values of *is* and *ds*.

When creating complex fixed point values, the fixed point representation can be different for the real and imaginary parts. In this case *is* and *ds* are complex integers. Additionally the maximum value of the sum of *is* and *ds* is limited by the representation of long integers to either 30 or 62.

Called with only two arguments, the fixed point variable that is created will contain only zeros. A third argument can be used to give the values of the fixed variables elements. This third argument *f* can be either a fixed point variable itself, which results in a new fixed point variable being created with a different representation, or a real or complex matrix.

4.2.21 fixed_inc

y = **fixed_inc** (*x*) Loadable Function

Example code of the use of the fixed point types in an oct-file. Returns `x + 1`

4.2.22 fixed_point_count_operations

val = **fixed_point_count_operations** () Loadable Function
old_val = **fixed_point_count_operations** (*new_val*) Loadable Function

Query or set the internal variable `fixed_point_count_operations`. If enabled, Octave keeps track of how many times each type of floating point operation has been used internally. This can be used to give an approximation of the algorithms complexity. By default, this feature is disabled. See also: display_fixed_operations

4.2.23 fixed_point_debug

val = **fixed_point_debug** () Loadable Function
old_val = **fixed_point_debug** (*new_val*) Loadable Function

Query or set the internal variable `fixed_point_debug`. If enabled, Octave keeps a copy of the value of fixed point variable internally. This is useful for use with a debug to allow easy access to the variables value. By default this feature is disabled.

4.2.24 fixed_point_library_version

fixed_point_library_version () Loadable Function

A function returning the version number of the fixed point library used.

4.2.25 fixed_point_version

fixed_point_version () Loadable Function

A function returning the version number of the fixed point package used.

4.2.26 fixed_point_warn_overflow

val = **fixed_point_warn_overflow** () Loadable Function
old_val = **fixed_point_warn_overflow** (*new_val*) Loadable Function

Query or set the internal variable `fixed_point_warn_overflow`. If enabled, Octave warns of overflows in fixed point operations. By default, these warnings are disabled.

4.2.27 fixedpoint

fixedpoint (`'help'`) Function File
fixedpoint (`'info'`) Function File
fixedpoint (`'info'`, *mod*) Function File
fixedpoint (`'test'`) Function File
fixedpoint (`'test'`, *mod*) Function File

Manual and test code for the Octave Fixed Point toolbox. There are 5 possible ways to call this function

`fixedpoint ('help')`
: Display this help message. Called with no arguments, this function also displays this help message

`fixedpoint ('info')`
: Open the Fixed Point toolbox manual

`fixedpoint ('info',` *mod*`)`
: Open the Fixed Point toolbox manual at the section specified by *mod*

`fixedpoint ('test')`
: Run all of the test code for the Fixed Point toolbox *mod*

Valid values for the varibale *mod* are

'basics' The section describing the use of the fixed point toolbox within Octave

'programming'
The section descrining how to use the fixed-point type with oct-files

'example' The section describing an in-depth example of the use of the fixed-point type

'reference' The refernce section of all of the specific fixed point operators and functions

Please note that this function file should be used as an example of the use of this toolbox

4.2.28 float

y = **float** (*x*) Function File

Converts a fixed point object to the equivalent floating point object. This is equivalent to `x.x` if `isfixed(x)` returns true, and returns *x* otherwise

4.2.29 flog

y = **flog** (*x*) Loadable Function

Compute the natural logarithm of the fixed point value *x*.

See also: fexp, flog10, fpow

4.2.30 flog10

y = **flog10** (*x*) Loadable Function

Compute the base-10 logarithm of the fixed point value *x*.

See also: fexp, flog, fpow

4.2.31 fprod

y = **fprod** (*x*, *dim*) Loadable Function

Product of elements along dimension *dim*. If *dim* is omitted, it defaults to 1 (column-wise products).

See also: fsum, fsumsq

4.2.32 freal

y = **freal** (*x*) Loadable Function

Returns the real part of the fixed point value *x*.

4.2.33 freshape

freshape (*a*, *m*, *n*) Loadable Function

Return a fixed matrix with *m* rows and *n* columns whose elements are taken from the fixed matrix *a*. To decide how to order the elements, Octave pretends that the elements of a matrix are stored in column-major order (like Fortran arrays are stored).

For example,

```
freshape (fixed(3, 2, [1, 2, 3, 4]), 2, 2)
ans =

  1.00  3.00
  2.00  4.00
```

If the variable `do_fortran_indexing` is nonzero, the `freshape` function is equivalent to

```
retval = fixed(0,0,zeros (m, n));
retval (:) = a;
```

but it is somewhat less cryptic to use `freshape` instead of the colon operator. Note that the total number of elements in the original matrix must match the total number of elements in the new matrix.

See also: ‘:’ and do_fortran_indexing

4.2.34 fround

y = **fround** (*x*) Loadable Function

Return the rounded value to the nearest integer of *x*.

See also: ffloor, fceil

4.2.35 fsin

y = **fsin** (*x*) Loadable Function

Compute the sine of the fixed point value *x*.

See also: fcos, fcosh, fsinh, ftan, ftanh

4.2.36 fsinh

y = **fsinh** (*x*) Loadable Function

Compute the hyperbolic sine of the fixed point value *x*.

See also: fcos, fcosh, fsin, ftan, ftanh

4.2.37 fsort

[*s*, *i*] = **fsort** (*x*) Function File

Return a copy of the fixed point variable *x* with the elements arranged in increasing order. For matrices, `fsort` orders the elements in each column

For example,

```
fsort (fixed(4,0,[1, 2; 2, 3; 3, 1]))
⇒  1  1
2  2
3  3
```

The `fsort` function may also be used to produce a matrix containing the original row indices of the elements in the sorted matrix. For example,

```
[s, i] = sort ([1, 2; 2, 3; 3, 1])
⇒ s = 1  1
2  2
3  3
⇒ i = 1  3
2  1
3  2
```

4.2.38 fsqrt

y = **fsqrt** (*x*) Loadable Function

Compute the square-root of the fixed point value *x*.

4.2.39 fsum

y = **fsum** (*x*, *dim*) Loadable Function

Sum of elements along dimension *dim*. If *dim* is omitted, it defaults to 1 (column-wise sum).

See also: fprod, fsumsq

4.2.40 fsumsq

y = **fsumsq** (*x*, *dim*) Loadable Function

Sum of squares of elements along dimension *dim*. If *dim* is omitted, it defaults to 1 (column-wise sum of squares). This function is equivalent to computing

```
fsum (x .* fconj (x), dim)
```

but it uses less memory and avoids calling `fconj` if *x* is real.

See also: fprod, fsum

4.2.41 ftan

y = **ftan** (*x*) Loadable Function

Compute the tan of the fixed point value *x*.

See also: fcos, fcosh, fsinh, ftan, ftanh

4.2.42 ftanh

y = **ftanh** (*x*) Loadable Function

Compute the hyperbolic tan of the fixed point value *x*.

See also: fcos, fcosh, fsin, fsinh, ftan

4.2.43 isfixed

isfixed (*expr*) Loadable Function

Return 1 if the value of the expression *expr* is a fixed point value.

4.2.44 lookup_table

y = **lookup_table** (*table*, *x*) Function File

y = **lookup_table** (*table*, *x*, *interp*, *extrap*) Function File

Using the lookup table created by *create_lookup_table*, find the value *y* corresponding to *x*. With two arguments the lookup is done to the nearest value below in the table less than the desired value. With three arguments a simple linear interpolation is performed. With four arguments an extrapolation is also performed. The exact values of arguments three and four are irrelevant, as only there presence detremines whether interpolation and/or extrapolation are used

4.2.45 reset_fixed_operations

reset_fixed_operations ( ) Loadable Function

Reset the count of fixed point operations to zero.

See also: fixed_point_count_operations, display_fixed_operations
